# Efficacy and safety of different medications compared for the treatment of postherpetic neuralgia: a network meta-analysis

**DOI:** 10.3389/fphar.2025.1614587

**Published:** 2025-07-30

**Authors:** Zhen Guo, Yunfan Xia, Zuyong Zhang

**Affiliations:** ^1^ The Fourth Clinical Medical College of Zhejiang Chinese Medical University, Hangzhou, Zhejiang, China; ^2^ The Third Clinical Medical College of Zhejiang Chinese Medical University, Hangzhou, Zhejiang, China; ^3^ Hangzhou Third People’s Hospital Affiliated to Zhejiang Chinese Medical University, Hangzhou, China

**Keywords:** postherpetic neuralgia, herpes zoster, network meta-analysis, randomized controlled trial, drug thearpy

## Abstract

**Background:**

Postherpetic neuralgia (PHN) is a neuropathic pain and the most common complication of herpes zoster (HZ). Pharmacotherapy serves as the primary intervention for alleviating pain associated with PHN.

**Methods:**

Electronic databases were systematically searched to identify randomized controlled trials (RCTs) evaluating pharmacotherapy for PHN. The network meta-analysis (NMA) based on the Bayesian framework was analyzed using R4.4.1 and Stata18.0 software.

**Results:**

A total of 38 RCTs were included in the analysis, enrolling 8,621 participants. In the Risk of Bias 2.0 (RoB 2.0) tool, nine studies (24%) were assessed as having a high risk of bias, 15 studies (39%) were rated as having some concerns, and 14 studies (37%) were assessed as having a low risk of bias. The NMA results showed that the NGX-4010 8% capsaicin patch had a statistically significant effect in terms of pain intensity (MD = −9.20, 95% CI: [−12.0, −6.60]). The secondary outcomes showed a significant effect of hydromorphone in improving sleep quality (MD = −3.8, 95% CI: [−23.0, −15.0]) and decreasing pain questionnaire scores (MD = −13.0, 95% CI: [−28.0, 2.1]). Amitriptyline plus pregabalin demonstrated the highest probability of clinical superiority (SUCRA = 0.92). The AE incidence results showed that opioids were identified as having the highest cumulative ranking (SUCRA = 0.87).

**Conclusion:**

The study showed that capsaicin patches and hydromorphone were more significant in relieving pain in PHN, whereas calcium channel modulators were more comprehensive in clinical management. The inclusion of more high-quality articles was needed to support this evidence due to quality bias in the literature.

## 1 Introduction

Postherpetic neuralgia (PHN) is a pathological neuralgia that results from direct damage to peripheral nerves during an HZ infection (Johnson and Rice, 2014). PHN is recognized as the most prevalent complication of HZ, primarily defined as persistent neuropathic pain lasting ≥3 months following lesion resolution (Forbes et al., 2016; Fornasari et al., 2022). The predominant pain characteristics observed in clinical settings are described as burning sensations, lancinating pain, or stabbing pain, accompanied by hyperalgesia and allodynia in the affected dermatomes, which are pathognomonic manifestations of neuropathic sensitization mechanisms (Schutzer-Weissmann and Farquhar-Smith, 2017). The severity of neuropathic pain has been observed to range from mild to severe, with manifestations categorized as persistent or intermittent patterns. These pain phenotypes have been significantly associated with the development of depressive symptoms, persistent fatigue, and sleep architecture disruption (Nahm et al., 2013; Colloca et al., 2017). From an epidemiological perspective, the global incidence of HZ has been established to range between three and five per 1,000 person-years, with progression to PHN documented in 5% to over 30% of HZ cases (Kawai et al., 2014). Moreover, a progressive elevation in PHN incidence has been epidemiologically correlated with advancing age (Sun et al., 2021; Gross et al., 2020). PHN prevalence rates reach as high as 75% in patients aged ≥70 years following acute HZ reactivation (Schmader, 2002). However, this severe chronic neuropathic pain syndrome has been recognized as imposing a substantial socioeconomic burden on healthcare systems globally (Schmader, 2002; Johnson et al., 2010; Corcuera-Munguia et al., 2023; Yin et al., 2021; Cuenca-Zaldívar et al., 2025). Clinically, the syndrome is accompanied by a significant deterioration in quality of life scores (Colloca et al., 2017). Therefore, the selection of therapeutic interventions for PHN is considered critically significant.

A wide variety of drugs are currently used to treat PHN, primarily calcium channel modulators (pregabalin, gabapentin), tricyclic antidepressants (TCAs), and 5% lidocaine medicated plasters (Finnerup et al., 2015). However, it has been demonstrated in previous studies that therapeutic interventions for PHN have been evaluated solely through direct comparisons or conventional meta-analyses (Han et al., 2013; Chen et al., 2014; Kim et al., 2017), thereby restricting the ability of clinicians to formulate evidence-based therapeutic strategies based on hierarchical efficacy rankings.

The NMA is conducted by integrating both direct and indirect evidence from existing RCTs, thereby providing hierarchical rankings of various interventions based on the Surface Under the Cumulative Ranking Area (SUCRA). Concurrently, transitivity is quantified through node-splitting tests to validate the consistency of evidence synthesis (Jansen and Naci, 2013), thereby establishing evidence-based therapeutic strategies for PHN.

Therefore, this study was designed to systematically compare the efficacy and safety of pharmacological interventions for PHN through a Bayesian framework-based NMA, which integrates clinical data from previous RCTs. The hierarchical differences between therapeutic agents were comprehensively analyzed to quantify comparative advantages in pain reduction, functional improvement, and safety profiles, thereby generating evidence-based recommendations for optimizing clinical decision-making in PHN management.

## 2 Methods

### 2.1 Protocol

This study was conducted by the guidelines of the Cochrane Handbook for Systematic Reviews of Interventions and the Preferred Reporting Items for Systematic Reviews and Meta-Analyses (PRISMA) statement (Cumpston et al., 2019; Shamseer et al., 2015; Hutton et al., 2015). The detailed protocol was prospectively registered in the international PROSPERO (registration number: CRD420250651348). Ethical approval and informed consent were not required because the analysis was based on previously published clinical studies.

### 2.2 Eligibility criteria

This study strictly adhered to the PICOS framework to define inclusion criteria (Izurieta et al., 2021): (1) Participants: Eligible patients were diagnosed with PHN, defined by the American Academy of Family Physicians (AAFP) as persistent dermatomal pain lasting 30 days to 6 months or longer after lesion resolution. No restrictions were applied to age, sex, nationality, or other demographic factors. (2) Intervention: This study focuses on pharmacological interventions for pain management in PHN patients, primarily examining first-line therapeutic agents, including pregabalin, gabapentin, opioid analgesics, and topical capsaicin patch. (3) Comparators: Control groups were defined as receiving either placebo treatment or standard pharmacological interventions. (4) The primary outcomes encompassed pain intensity and analgesic response, evaluated through validated instruments, including the Visual Analog Scale (VAS), Numeric Rating Scale (NRS), and Average Daily Pain Score (ADPS). Secondary outcomes comprised the Short-Form McGill Pain Questionnaire (SF-MPQ) and the Pittsburgh Sleep Quality Index (PSQI). Pain relief was judged by pain relief rate and clinical effectiveness rate. (5) Study designs: This study included only randomised controlled trials of the drug therapy for PHN.

### 2.3 Data sources and search strategy

Searches of the electronic literature were conducted in five major databases: The Cochrane Library, Web of Science, PubMed, MEDLINE, and Embase. The search strategy was implemented without restrictions on language, country of origin, or publication type. The retrieval timeframe spanned from the inception of each database to March 2025, ensuring maximal coverage of both historical and contemporary evidence. The search terms were “Neuralgia, Postherpetic,” “Herpes Zoster,” “Drug therapy,” and “randomized controlled trial.” The full literature search strategy is detailed in Supplementary Material S1.

### 2.4 Screening process

Two reviewers (GZ and XYF) independently screened the literature in the database based on predefined inclusion criteria. The retrieved bibliographic records were imported into NoteExpress, and duplicates were removed. Two reviewers (GZ and XYF) independently screened the titles and abstracts of the retrieved literature. At this stage, studies deemed irrelevant to the study objectives were excluded. The full text of the remaining articles was then further assessed for eligibility for inclusion in the NMA. Disagreements between the two reviewers were initially resolved through structured discussions. If consensus could not be reached, unresolved issues were referred to a third independent reviewer (ZZY) for arbitration until the three reviewers agreed.

### 2.5 Data extraction

Two reviewers (GZ and XYF) independently performed data extraction and extracted the following information: (1) General information: title, author, year of publication; (2) Study designs: sample size, randomisation, blinding, number of study groups, study duration, number of RCTs enrolled; (3) Intervention and control: drug therapy and control group; (4) Outcomes: primary and secondary outcome indicators, AEs, clinical effectiveness and conclusions.

### 2.6 Risk of bias assessment

The methodological quality of each included study was independently assessed using the Cochrane RoB 2.0 tool (Sterne et al., 2019), focusing on five critical domains: randomisation process, deviations from intended interventions, missing outcome data, measurement of the outcome, and selection of the reported result. This evaluation aimed to characterize potential biases as “low risks,” “some concerns,” or “high risks” based on predefined signaling questions and decision algorithms outlined in the tool. Two reviewers (GZ and XYF) independently evaluated each study for bias, resolving disagreements by consulting a third reviewer (ZZY).

### 2.7 Statistical analysis

Bayesian NMA was conducted using the GeMTC package (version 1.6-2) within the R Studio environment (version 4.4.1), and network evidence was mapped using STATA (18.0). Bayesian NMA under a random-effects model with vague prior information was implemented using Markov chain Monte Carlo (MCMC) methods. Four parallel MCMC chains were simultaneously initiated, with 50,000 iterations per chain (20,000 burn-in iterations for adaptation and 30,000 posterior sampling iterations) to ensure convergence and minimize autocorrelation. Pooled analyses of all outcomes were performed using a random-effects model to account for inter-study heterogeneity. For continuous variables, treatment effects were expressed as mean difference (MD), whereas dichotomous outcomes were analysed as odds ratios (OR). 95% confidence intervals (CI) were used to analyse all data. Trace and kernel density plots were used to assess the convergence of the data, and sorted plots of the SUCRA were used to assess each outcome indicator for each intervention, with larger SUCRA values indicating that the treatment program was more effective. Model inconsistency was evaluated using the node-splitting method, which assesses discrepancies between direct and indirect evidence within closed-loop network structures. Statistical heterogeneity was quantified by the I^2^ statistic and Cochran’s Q test (threshold: I^2^ > 50%, p < 0.05), indicating substantial heterogeneity that required further heterogeneity analysis. An assessment of publication bias in outcome indicators by plotting funnel plots. Funnel plot asymmetry was further evaluated using Egger’s linear regression test (P < 0.05 indicating significant bias). A symmetrical distribution of effect estimates clustered uniformly around the null value (X = 0) was interpreted as no significant evidence of publication bias or small-study effects.

## 3 Results

### 3.1 Literature search

**FIGURE 1:**
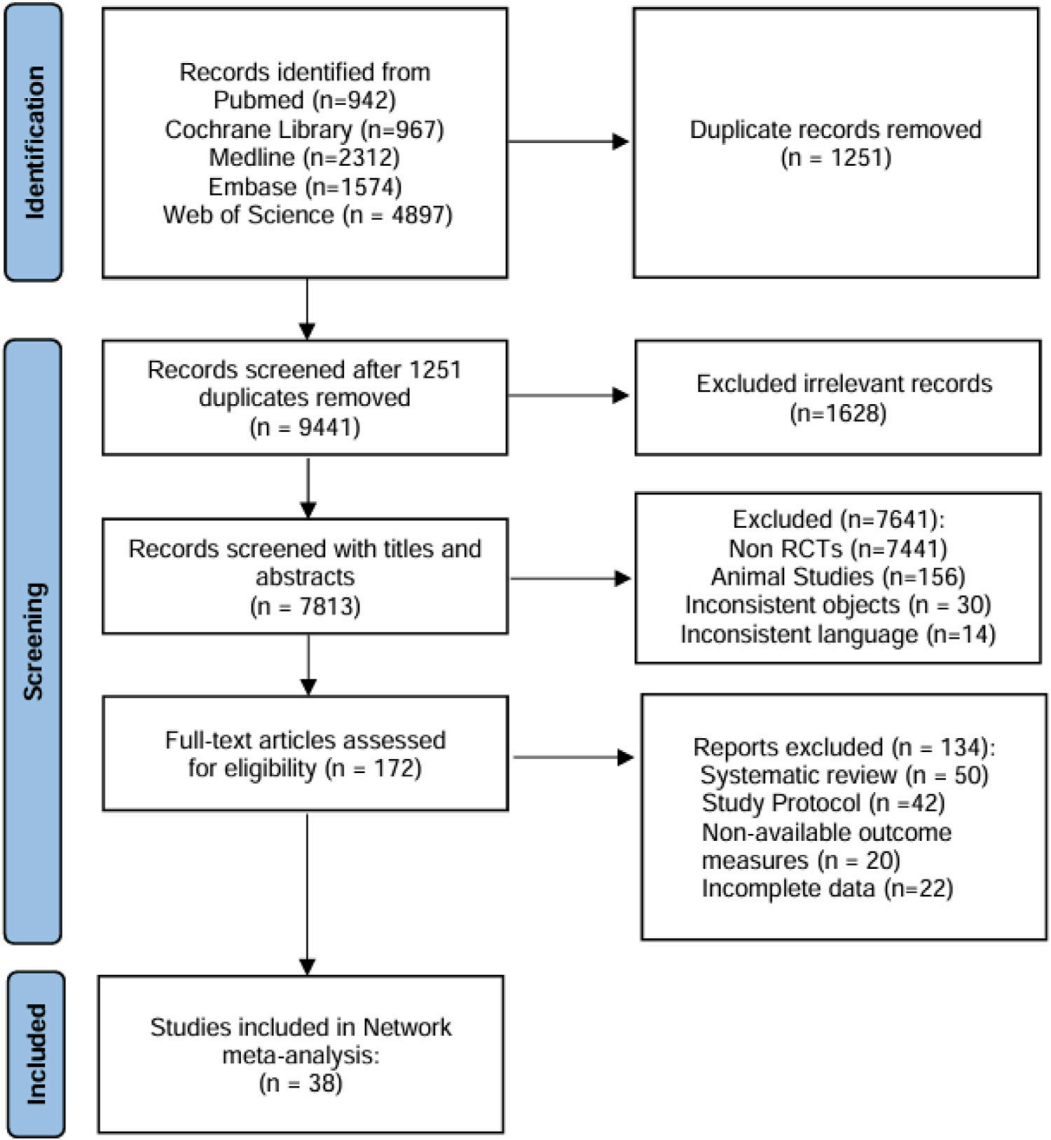
Flow chart of study selection.

A total of 10,692 references were retrieved from five electronic databases, and 1,251 duplicates were removed. Subsequent title and abstract screening excluded 7,641 records deemed irrelevant to neuropathic pain interventions or lacking comparative effectiveness data for PHN therapies. After two reviewers (GZ and XYF) assessed the eligibility of full-text articles, 38 references were finally included for the NMA (Figure 1).

### 3.2 Study characteristics

A total of 38 studies were included in the analysis, with 26 two-arm studies, nine three-arm studies, and three four-arm studies. The included studies were published over a time span of 2001-2024 and enrolled 8,621 participants with treatment durations ranging from 7 days to 6 months. In this study, the references included were both RCTs, and the specific characteristics of the references were shown in Table 1.

**TABLE 1:** Characteristics of studies selected for NMA.

References	Type of study	Total patients	Intervention	Control	Primary outcomes	Treatment period	Adverse events
Rice (2001)	RCTs, double-blind	334	Gabapentin 1,800 mg/2,400 mg/day	Placebo	Average daily pain score	7 weeks	Dizziness, somnolence
Raja et al. (2002)	RCTs, double-blind, crossover study	76	Opioids 91 mg/day, TCA 89 mg/day	Placebo	Pain intensity ratings	24 weeks	Constipation, nausea
Dworkin et al. (2007)	RCTs, double-blind	173	Pregabalin 600 mg/300 mg/day	Placebo	Pain scores	8 weeks	Dizziness, somnolence
Boureau et al. (2003)	RCTs, double-blind	127	Tramadol 100 mg/day	Placebo	VAS	6 weeks	Nausea
Sabatowski et al. (2004)	RCTs, double-blind	238	Pregabalin 150 mg/300 mg/day	Placebo	NPRS	8 weeks	Dizziness, somnolence
Rowbotham et al. (2005)	RCTs, double-blind	47	Desipramine/Amitriptyline 150 mg/day	Fluoxetine 60 mg/day	VAS	9 weeks	Dry mouth, constipation
van Seventer et al. (2006)	RCTs, double-blind	370	Pregabalin 150 mg/300 mg/600 mg/day BID	Placebo	NPRS	13 weeks	Dizziness, somnolence
Backonja et al. (2008)	RCTs, double-blind	402	NGX-4010, 8% capsaicin patch/60-min	0.04% capsaicin patch/60-min	NPRS	12 weeks	Erythema, pain
Binder et al. (2009)	RCTs, double-blind	71	5% lidocaine medicated plasters/12 h per day	Placebo plaster	VRS	2 weeks	Skin and subcutaneous tissue disorders
Jensen et al. (2009)	RCTs, double-blind	158	Extended-release gabapentin 1,800 mg/day	Placebo	NPRS	4 weeks	NA
Shackelford et al. (2009)	RCTs, double-blind	209	GW406381 25 mg/30 mg/day	Placebo	NPRS	3 weeks	Headache, diarrhea
Kanai et al. (2009)	RCTs, double-blind, crossover study	24	8% lidocaine	Placebo	VAS	7 days	NA
Wallace et al. (2010)	RCTs, double-blind	407	Extended-release gabapentin 1,800 mg/BID/QD	Placebo	ADPS	10 weeks	Nervous system disorders
Rehm et al. (2010)	RCTs, open-label	98	5% lidocaine medicated plaster/day/TID	Pregabalin 150/300 mg/day	PPS	12 weeks	Dizziness, fatigue
Webster et al. (2010a)	RCTs, double-blind	155	NGX-4010, 8% capsaicin patch/60-min	0.04% capsaicin patch/60-min	NPRS	12 weeks	Pruritus, dryness
Webster et al. (2010b)	RCTs, double-blind	299	NGX-4010, 8% capsaicin patch/60-min	0.04% capsaicin patch/60-min	NPRS	12 weeks	Application site reactions
Backonja et al. (2010)	RCTs, open-label, double-blind	38	NGX-4010, 8% capsaicin patch/60-min	0.04% capsaicin patch/60-min	NPRS	4 weeks	Dizziness, fatigue
Backonja et al. (2011)	RCTs, double-blind	101	Gabapentin enacarbil 1,200 mg/day/BID	Placebo	VAS	14 days	Dizziness, nausea
Irving et al. (2011)	RCTs, double-blind	418	NGX-4010, 8% capsaicin patch/60-min	0.04% capsaicin patch/60-min	NPRS	12 weeks	Application site erythema
Achar et al. (2010)	RCTs	45	Amitriptyline 25 mg/day/QD, Pregabalin 75 mg/day/BID	Amitriptyline plus pregabalin/day	Pain relief ratings	8 weeks	Dizziness, dryness, drowsiness
Gu et al. (2012)	RCTS	52	Pregabalin 150 mg/day	Routine drug treatment	NPRS	4 weeks	Dizziness, somnolence
Irving et al. (2012)	RCTs, double-blind	1,127	NGX-4010, 8% capsaicin patch/60-min	0.04% capsaicin patch/60-min	NPRS	12 weeks	Erythema, pain
Apalla et al. (2013)	RCTs, double-blind	30	Botulinum toxin A/100 IU/day	Placebo	VAS	4 weeks	NA
Zhang et al. (2013)	RCTs, double-blind	376	Gabapentin enacarbil 1,200 mg/2,400 mg/3,600 mg/day	Placebo	NPRS	14 weeks	Dizziness, somnolence
Achar et al. (2013)	RCTs, open-label	50	Amitriptyline 25 mg/day/QD	Pregabalin 75 mg/day/BID	categorical scale	6 months	Dizziness, dryness
Freeman et al. (2015)	RCTs, double-blind	719	Gastroretentive gabapentin 1,800 mg/day	Placebo	BPI/NPRS	10 weeks	NA
Gavin et al. (2017)	RCTs, double-blind	28	TPM/oxycodone patch/72 h	Vehicle patch/72 h	NPRS	17 days	Application site irritation
Liu et al. (2017)	RCTs, double-blind	220	Pregabalin 300 mg/day	Placebo	DPRS	8 weeks	Dizziness, peripheral edema
Liu et al. (2018)	RCTs, double-blind	183	5 mg/kg intravenous lidocaine infusion	Placebo	VAS	4 weeks	Dizziness, dry mouth
Kato et al. (2019)	RCTs, double-blind	765	Mirogabalin 15 mg/20 mg/30 mg/day	Placebo	ADPS	14 weeks	Dizziness, somnolence
Bulilete et al. (2019)	RCTs, double-blind	98	Gabapentin 1,800 mg/day	Placebo	VAS	5 weeks	NA
Khajuria et al. (2021)	RCTs, open-label	48	Pregabalin 150 mg/day	Nortriptyline 25 mg/day	NPRS	8 weeks	Dizziness, dry mouth
Huang et al. (2021)	RCTs	201	Pregabalin 75 mg and IV PCA hydromorphone 2 mg/day	Pregabalin 75 mg/day	NPRS	2 weeks	Dizziness, nausea
Jin et al. (2021)	RCTs, double-blind	75	Bulleyaconitine with gabapentin 0.4 mg/day/TID	Placebo	VAS	4 weeks	Dizziness, nausea
Sun et al. (2021)	RCTs, double-blind	80	Epidural morphine 5 mg/day	Epidural hydromorphone 1 mg/day	VAS	3 months	Nausea, vomiting
Wang et al. (2023)	RCTs, double-blind	240	5% lidocaine medicated plaster/day	Placebo	VAS	4 weeks	Skin disease
Kawai et al. (2014)	RCTs, double-blind	173	Tramadol 400 mg/day	Placebo	NPRS	4 weeks	Nausea, constipation
Zhang et al. (2024)	RCTs, double-blind	366	Crisugabalin 40 mg/80 mg/day	Placebo	ADPS	12 weeks	Dizziness, hyperuricemia

### 3.3 Risk of bias results

**FIGURE 2:**
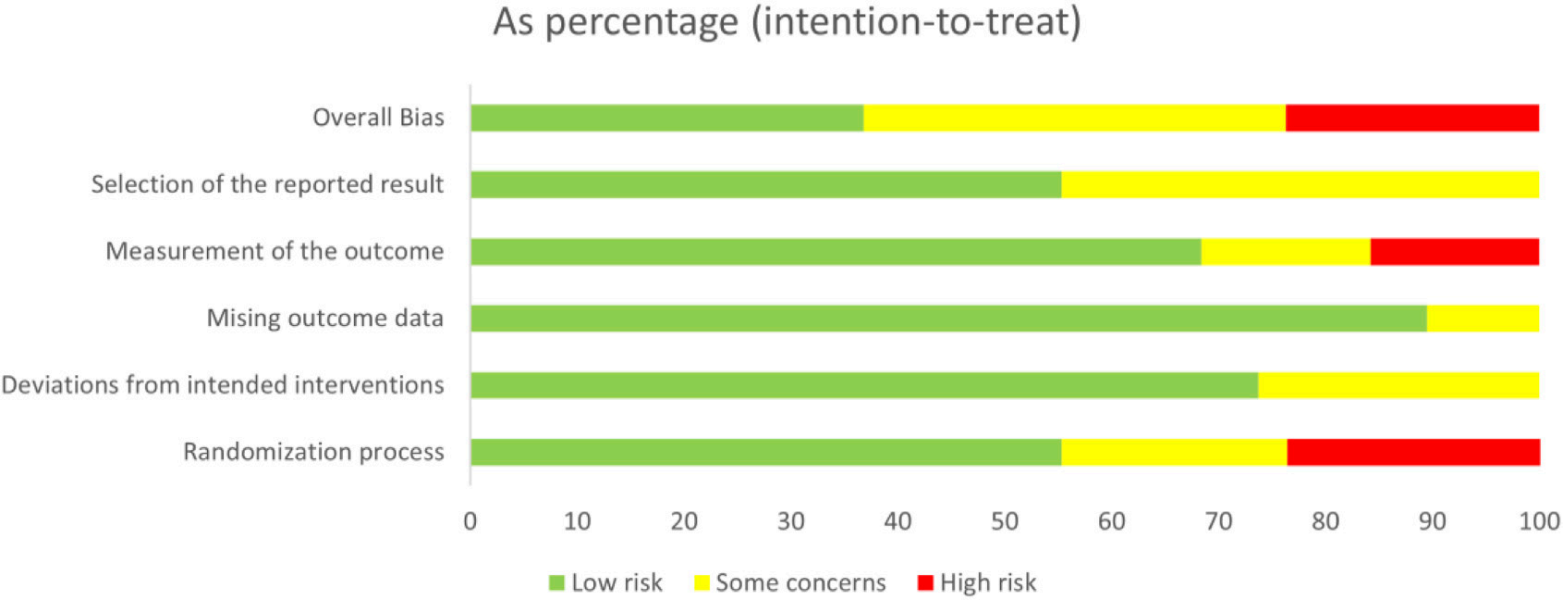
The summary by the cochrane risk of bias tool.

Among the 38 included RCTs, nine studies (24%) were assessed as having a high risk of bias, primarily attributed to unclear randomization processes, e.g., flaws in randomization, lack of blinding could lead to exaggerated efficacy, resulting in a spurious upgrading of the drug in the SUCRA rankings, and thus affecting the reliability of the SUCRA rankings. 15 studies (39%) were rated as having some concerns, primarily attributed to unclear outcome measurement protocols and the selective reporting of results. For example, the unpublished of negative results may have led to an underestimation of the placebo effect and thus a lower SUCRA ranking. 14 studies (37%) were assessed as having a low risk of bias. The results of the quality assessment of all studies were illustrated in Figure 2, which showed an overview of the judgments for each risk of bias item, indicated as a percentage of all included studies, and revealed a summary of the risk of bias by the two reviewers (GZ and XYF).

### 3.4 Statistical analysis results

#### 3.4.1 Model convergence evaluation result

**FIGURE 3:**
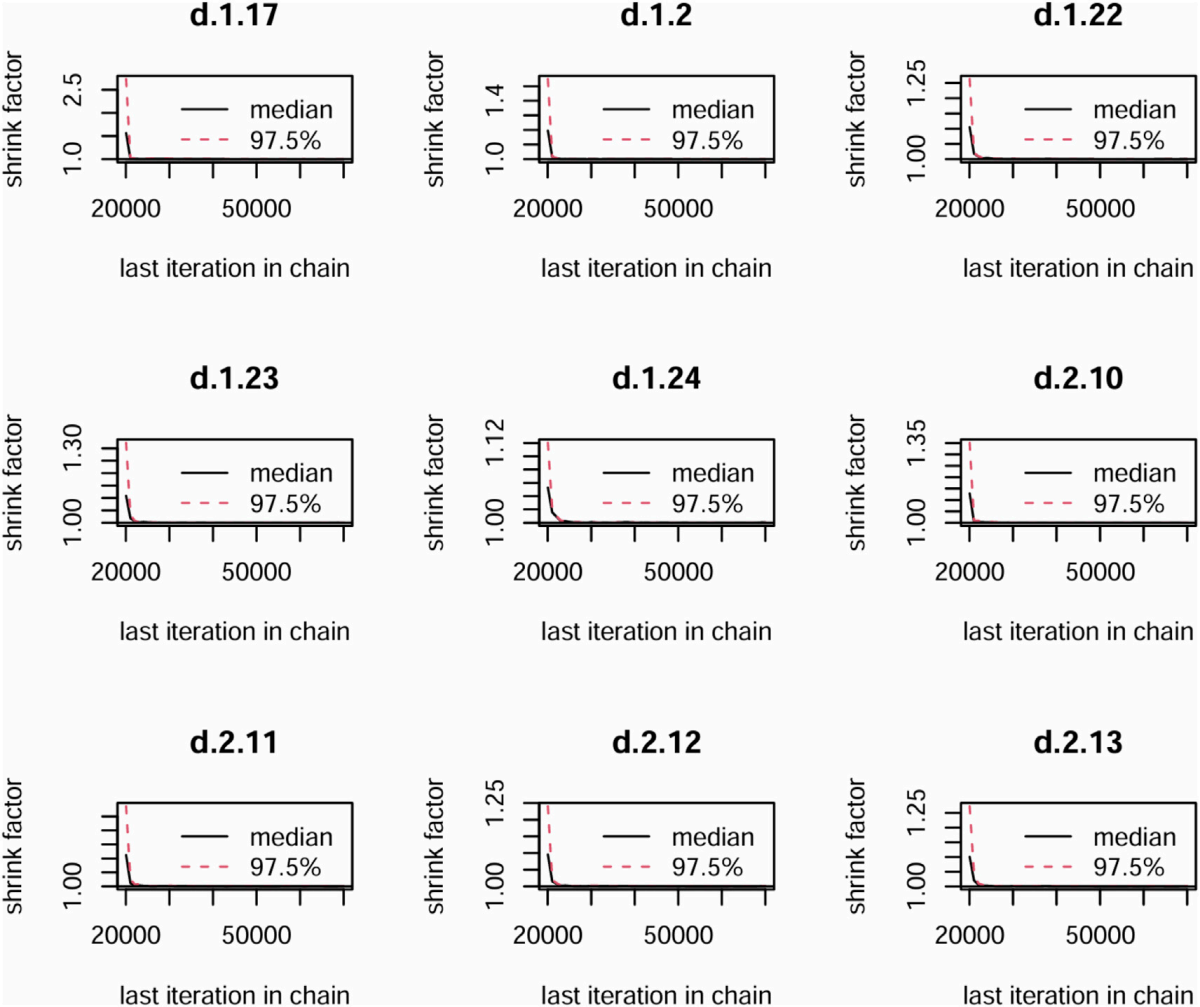
The Primary outcome convergence diagnostic map. 1 = Pregabalin, 2 = Placebo, 17 = 5% lidocaine medicated plasters, 22 = hydromorphone, 23 = Routine drug treatment, 24 = Nortriptyline, 10 = 8% lidocaine, 11 = TCAs, 12 = Gabapentin Enacarbil 1,200 mg, 13 = Gabapentin Enacarbil 2,400 mg.

In the Bayesian framework constructed for this study, all Markov chains achieved stable convergence after 50,000 iterations, with trace plots shown with stationary trajectories and minimal autocorrelation. The Brooks-Gelman-Rubin diagnostic results demonstrated satisfactory model convergence, as evidenced by the median and 97.5th percentile of the potential scale reduction factor (PSRF) stabilized toward 1.0 after 20,000–30,000 iterations, with both univariate PSRF and multivariate PSRF (mPSRF) values remaining below 1.05 (Figure 3). Trace and density plots demonstrated robust convergence characteristics, with bandwidth parameters asymptotically approaching 0 and stabilized after 50,000 iterations, indicating stationary posterior distributions across all Markov chains (Supplementary Material S2).

#### 3.4.2 Network evidence diagram

**FIGURE 4:**
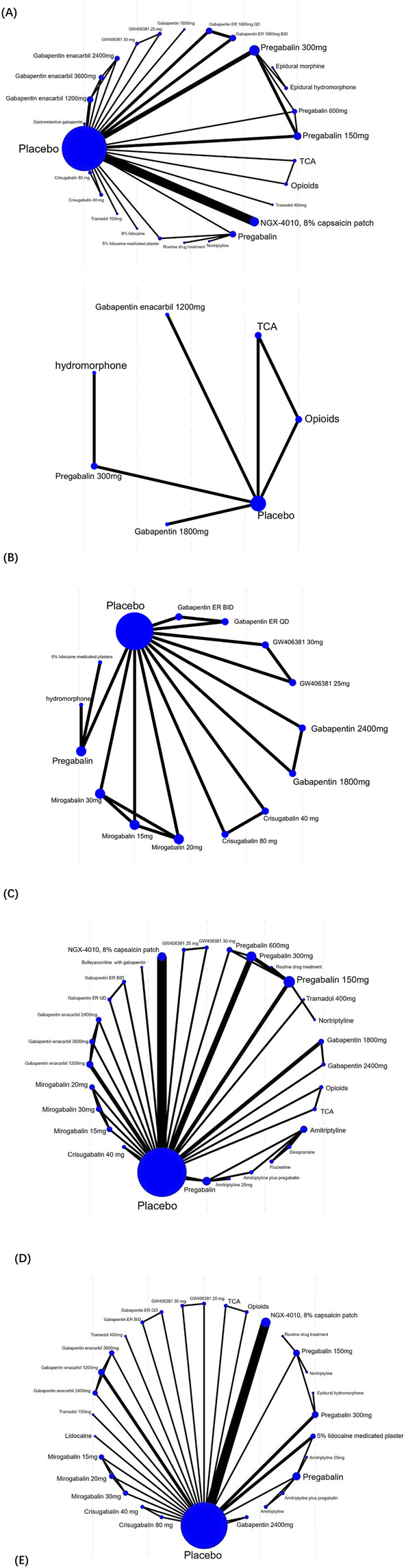
Network plot of included studies. (**(A)** for the pain intensity outcomes, **(B)** for the sleep quality outcomes, **(C)** for the SFMPQ outcomes, **(D)** for the clinical effective rates, **(E)** for the AEs).

The specific network evidence maps were created for the different outcome data (Figure 4). Node sizes were weighted to reflect the patient cohort size within each intervention, while the thickness of the line was proportionally scaled to the cumulative sample size of participants involved in pairwise comparisons. Pain intensity outcomes were reported in 28 studies and involved 27 treatment modalities, which formed 11 closed loops. The SFMPQ outcomes were reported by 8 RCTs, which encompassed 15 distinct therapeutic modalities, and seven closed-loop comparisons were generated through the synthesis of direct and indirect evidence. Sleep quality outcomes were reported by 5 RCTs, which included seven different treatment modalities. A single closed-loop comparison was formed through the synthesis of direct and indirect evidence among the placebo, TCAs, and opioids. Clinical effective rates were reported by 27 RCTs, which included 32 distinct therapeutic modalities. Six therapeutic modalities failed to form closed-loop comparisons due to insufficient direct or indirect evidence for Bayesian network meta-analyses. AEs were reported by 29 RCTs, which included 32 distinct therapeutic modalities; eight therapeutic modalities failed to form closed-loop comparisons.

#### 3.4.3 Network meta-analysis results of pain scores

**FIGURE 5:**
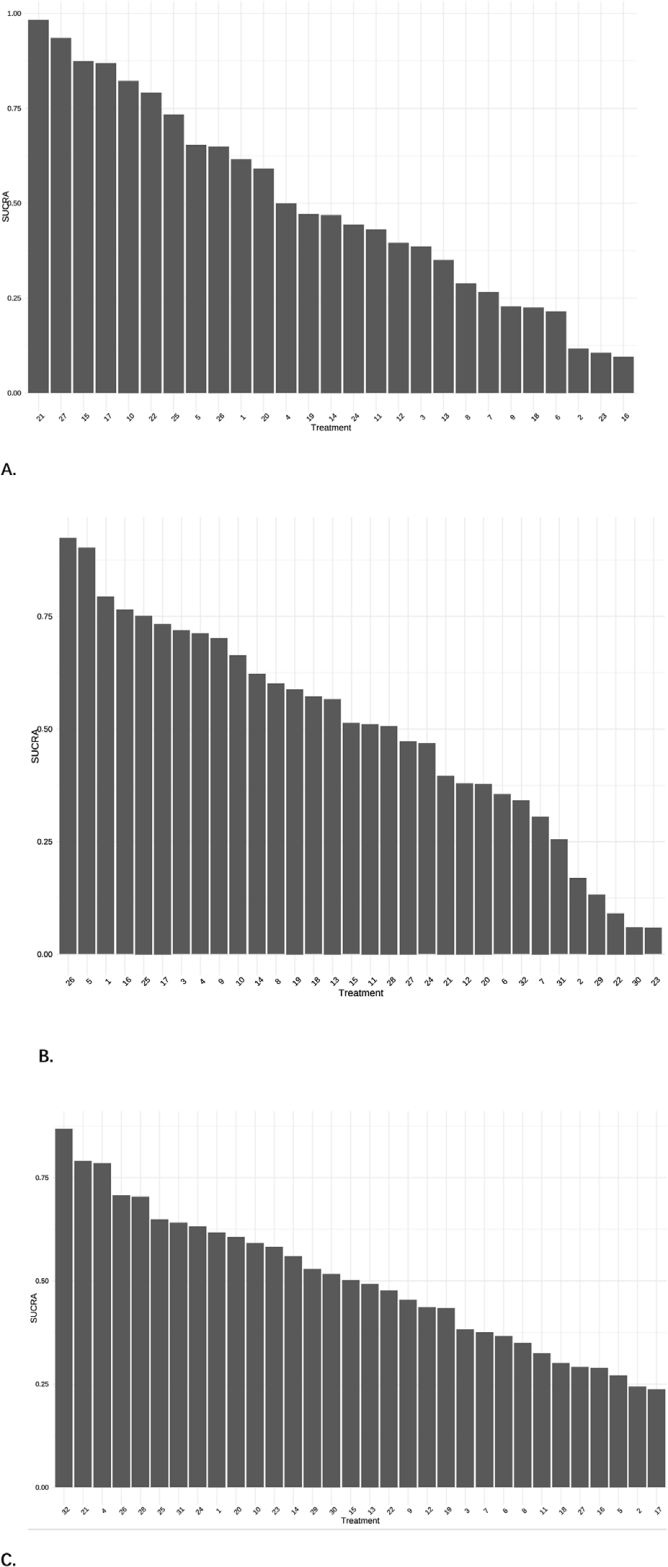
Cumulative probability plots. **(A)** The results of pain scores, 21 = NGX-4010, 8% capsaicin patch, 27 = Tramadol 100 mg, 15 = Gastroretentive Gabapentin. **(B)** The results of effective rate, 26 = Amitriptyline plus pregabalin, 5 = Pregabalin 600 mg, 1 = Pregabalin. **(C)** The results of AEs, 32 = Opioids, 21 = Mirogabalin 30 mg, 4 = Pregabalin 300 mg.

**FIGURE 6:**
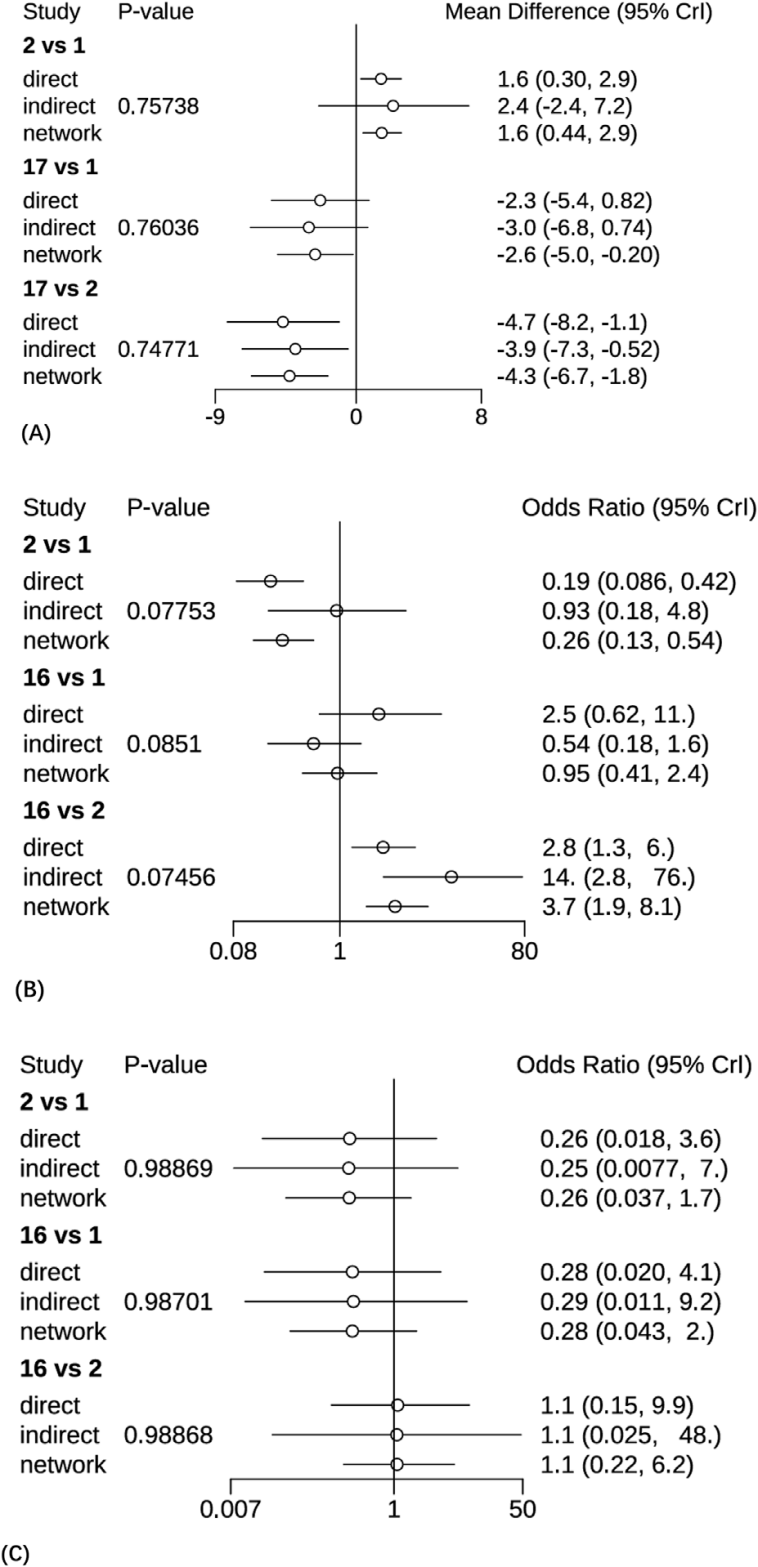
The Node-splitting methods for forest plots. **(A)** The pain intensity outcomes, 1 = Pregabalin, 2 = Placebo, 17 = 5% lidocaine medicated plasters. **(B)** The clinical effective rates, 1 = Pregabalin, 2 = Placebo, 16 = 5% lidocaine medicated plasters. **(C)** The AEs, 1 = Pregabalin, 2 = Placebo, 16 = 5% lidocaine medicated plasters.

A total of 28 studies encompassing 6,946 patients diagnosed with PHN were included in this NMA. Compared with placebo, no statistically significant differences were observed for routine drug treatment (MD = 0.35, 95% CI: [−1.4, 2.1]) and Gabapentin 1,800 mg (MD = 0.30, 95% CI: [−1.0, 1.6]) in pain reduction outcomes, whereas all other therapeutic modalities demonstrated statistically significant efficacy. Forest plots were detailed in Supplementary Material S3. A comprehensive pairwise comparison matrix was constructed to evaluate all therapeutic interventions (Supplementary Material S4). Statistically significant differences (P < 0.05) were identified in 29 pairwise comparisons of interventions, as evidenced by non-overlapping 95% CI in pain reduction outcomes. A statistically significant reduction in pain intensity was demonstrated for the NGX-4010 8% capsaicin patch (MD = −9.20, 95% CI: [−12.0, −6.60]). The pain score results for all interventions were ranked according to SUCRA values, and the complete ranked effects were shown in Figure 5, the NGX-4010 8% capsaicin patch was identified as demonstrating the highest probability of superior efficacy (SUCRA = 0.98), followed by tramadol 100 mg (SUCRA = 0.93) and gastroretentive gabapentin (SUCRA = 0.88). The Consistency evaluation was performed, and the deviance information criterion (DIC) values demonstrated the absence of global inconsistency (consistency model DIC = 125.6, UME model DIC = 127.39). Local inconsistency evaluation was conducted using the node-splitting method. No statistically significant differences were observed between direct and indirect evidence comparisons across all intervention nodes (P > 0.1), indicating robust consistency in the network topology (Figure 6). Heterogeneity testing was performed, and I^2^ = 41%, P > 0.1, which suggested that there was no significant heterogeneity in the interventions between groups.

#### 3.4.4 Network meta-analysis results of the effective rate

A total of 27 studies involving 6,864 patients were included in this analysis. All interventions demonstrated statistically significant efficacy when compared to placebo (Supplementary Material S3), except for routine drug treatment (OR = 0.39, 95% CI: [0.073, 1.8]), nortriptyline (OR = 0.19, 95% CI: [0.0063, 1.8]), amitriptyline (OR = 0.42, 95% CI: [0.040, 3.0]), and fluoxetine (OR = 0.17, 95% CI: [0.0096, 2.2]). A pairwise comparison matrix was generated (Supplementary Material S4), and statistically significant differences were found in the 131 intervention comparisons evaluated. The clinical effective rate of nortriptyline (−4.03, 95% CI: [−8.01, −1.14]) for the treatment of PHN was remarkable. The efficacy hierarchy of all interventions was ranked based on the SUCRA values (Figure 5). Amitriptyline plus pregabalin demonstrated the highest probability of clinical superiority (SUCRA = 0.92), followed by pregabalin 600 mg (SUCRA = 0.90) and standard-dose pregabalin (SUCRA = 0.79). The Consistency evaluation was performed, and DIC values demonstrated the absence of global inconsistency (consistency model DIC = 136.1, UME model DIC = 134.93). No statistically significant differences were observed between direct and indirect evidence comparisons across all intervention nodes (P > 0.05), indicating robust consistency in the network topology (Figure 6). Heterogeneity testing was performed, and I^2^ = 0%, P > 0.05, which suggested that there was no significant heterogeneity in the interventions between groups.

#### 3.4.5 Network meta-analysis results of SFMPQ scores

A total of eight studies involving 2,583 patients were included in this analysis. All interventions demonstrated statistically significant efficacy when compared to placebo (Supplementary Material S3). The results of SFMPQ for all interventions were ranked according to SUCRA values, and the complete ranked effects were shown in Supplementary Material S5. Hydromorphone was identified as demonstrating the highest probability of superior efficacy (SUCRA = 0.87), followed by 5% lidocaine medicated plasters (0.79) and Mirogabalin 30 mg (0.71). The Consistency evaluation was performed, and DIC values demonstrated the absence of global inconsistency (consistency model DIC = 44.08, UME model DIC = 44.06).

#### 3.4.6 Network meta-analysis results of PSQI scores

A total of five studies involving 678 patients were included in this analysis. All interventions demonstrated statistically significant efficacy when compared to placebo (Supplementary Material S3), except Gabapentin 1,800 mg (MD = 11.0, 95% CI: [−5.8, 27.0]) and Opioids (MD = 0.088, 95% CI: [−13.0, 14.0]). The results of sleep quality for all interventions were ranked according to SUCRA values, and the complete ranked effects were shown in Supplementary Material S5. The improvement in sleep quality was most prominently demonstrated by hydromorphone (0.75), followed by gabapentin enacarbil 1,200 mg (0.61) and TCA (0.53). The Consistency evaluation was performed, and DIC values demonstrated the absence of global inconsistency (consistency model DIC = 21.94, UME model DIC = 21.98).

#### 3.4.7 Network meta-analysis results of adverse events

**FIGURE 7:**
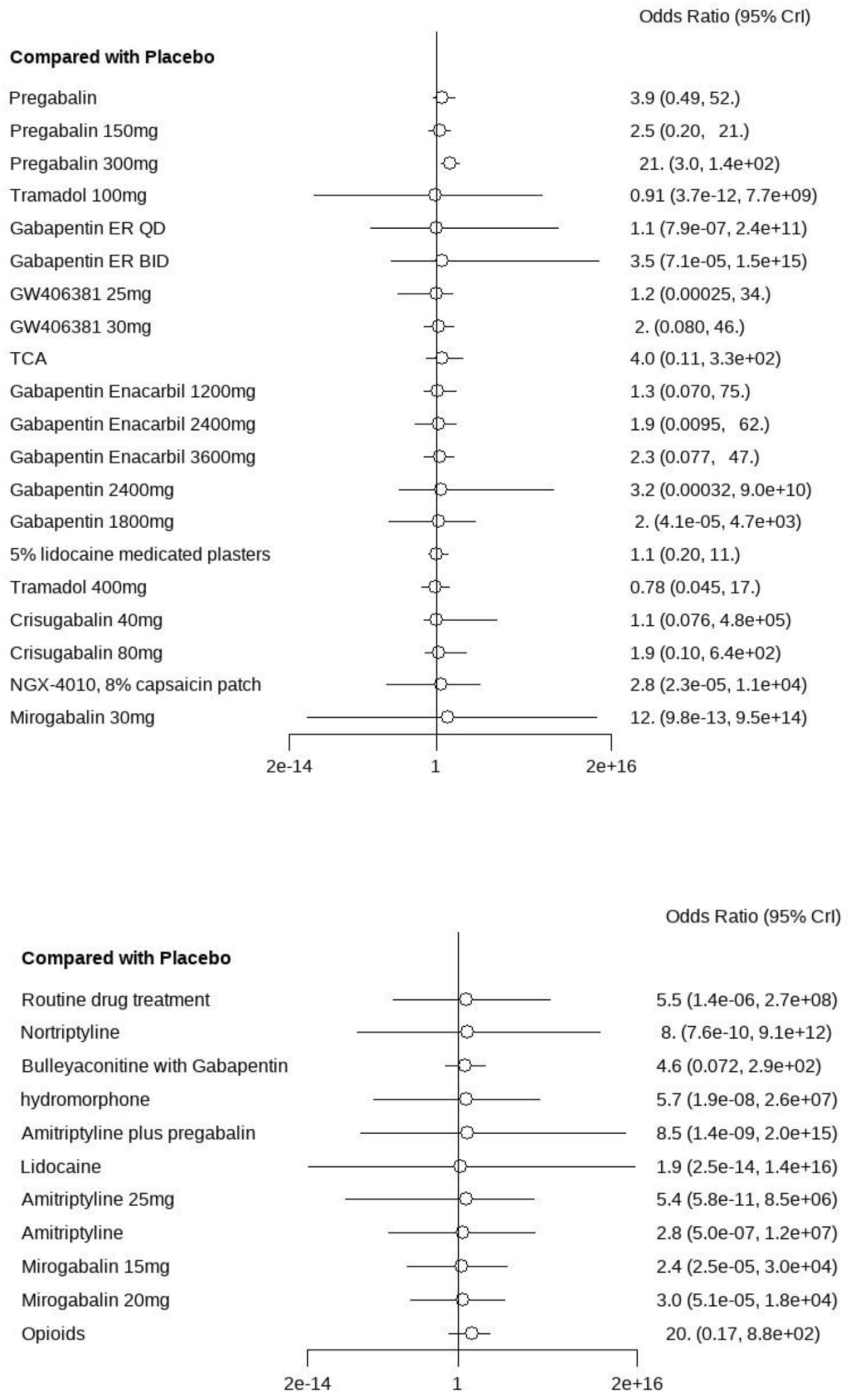
The Network meta-regression analysis results of adverse events.

A total of 29 studies encompassing 7,018 patients were systematically analyzed for AE incidence rates associated with pharmacological interventions. A significantly higher incidence of AEs was observed with opioids (OR = 20.0, 95% CI: [1.2, 4.3e+02]) compared to placebo, predominantly manifesting as constipation and nausea (Supplementary Material S3). No severe AEs were reported across the included studies. A pairwise comparison matrix was generated (Supplementary Material S4), and statistically significant differences were found in the 19 intervention comparisons evaluated. Opioids demonstrated a significantly higher incidence of AEs compared to other interventions (−3.28, 95% CI: [−7.13, 0.43]). The results of AE incidence rates for all interventions were ranked according to SUCRA values, and the complete ranked effects were shown in Figure 5. Opioids were identified as demonstrating the highest probability of superior efficacy (0.87), followed by Mirogabalin 30 mg (0.79) and Pregabalin 300 mg (0.78). The Consistency evaluation was performed, and DIC values demonstrated the absence of global inconsistency (consistency model DIC = 137.1, UME model DIC = 137.57). No statistically significant differences were observed between direct and indirect evidence comparisons across all intervention nodes (P > 0.05), indicating robust consistency in the network topology (Figure 6). Heterogeneity testing was conducted across intervention groups, these findings indicate significant heterogeneity in treatment effects between intervention arms (I^2^ pair = 84.01%, I^2^ cons = 64.27%). A network meta-regression analysis was conducted to explore sources of heterogeneity across intervention groups (Figure 7). The Rob assessment scores were incorporated into the model to evaluate their influence on the effect size (OR). Following the inclusion of Rob scores, the model heterogeneity was substantially reduced (I^2^ = 2%), indicating that methodological quality accounted for the majority of variability in the network. The model heterogeneity was substantially reduced (I^2^ = 2%), confirming that methodological quality stratification through Rob scoring was demonstrated to exert significant control over residual heterogeneity in the pooled estimates. However, the use of Rob as a covariate was mainly due to the significant impact of literature quality on heterogeneity. High risk of bias studies might exaggerate effect sizes, leading to increased heterogeneity, while low-quality studies might selectively report positive results, leading to a discrete distribution of effect sizes.

#### 3.4.8 Network meta-analysis results of publication bias

Publication bias and small sample effect for the primary outcomes, clinical response rates, and AEs incidences were assessed using comparative-adjusted funnel plots, as detailed in Supplementary Material S6. A statistically significant risk of publication bias or small sample effect was identified for the clinical response rate through Egger’s regression test (P < 0.01), suggesting potential compromise in the reliability of pooled estimates.

## 4 Discussion

A systematic comparison of therapeutic efficacy and safety profiles among drug interventions for PHN was conducted through Bayesian NMA. The evaluated interventions included calcium channel modulators (pregabalin, gabapentin), TCAs (amitriptyline), 5% lidocaine medicated plasters, opioids, and capsaicin patches.

Significant advantages in pain relief were demonstrated by the NGX-4010 8% capsaicin patch. This therapeutic effect is mechanistically attributed to capsaicin’s role as a selective agonist of the transient receptor potential vanilloid 1 (TRPV1) receptor, which induces nociceptor defunctionalization through sustained calcium influx-mediated depolarization (Bode and Dong, 2011; Derry et al., 2013). Subsequent action potential initiation and propagation along C-fibers are attenuated due to TRPV1 receptor desensitization, thereby interrupting peripheral pain signaling pathways (Bley, 2004)^.^ After sustained exposure to capsaicin, TRPV 1-containing sensory axons might enter a prolonged period of inactivity, blocking pain transmission and leading to a reduced pain response. NGX-4010 8% capsaicin patch was a highly concentrated capsaicin skin patch that had been proven to relieve pain (Backonja et al., 2010; Backonja et al., 2008; Irving et al., 2011). Hydromorphone showed significant benefits in improved sleep quality and decreased SFMPQ scores. PHN could last for weeks or longer, severely disrupting the sleep and daily life of the patient (Colloca et al., 2017; Johnson et al., 2010). Hydromorphone is classified as a semi-synthetic derivative of morphine, analgesic effects being mediated through selective agonism of mu-opioid receptors within the central nervous system (CNS) (Quigley, 2002). Although hydromorphone had been used to treat severe pain, its current use in PHN was less (Mahler and Forrest, 1975; Dworkin et al., 2007)^.^ Huang2021’s study concluded that hydromorphone treatment of PHN resulted in a significant decrease in PSQI scores and greater improvement in the sleep of patients (Huang et al., 2021). Amitriptyline plus pregabalin had the highest clinical efficacy rate, this finding highlighted that optimization strategies for combination therapies would be a priority in future research agendas (Gonzalez-Alvarez et al., 2023; Martín Pérez et al., 2023). However, Opioids had the highest incidence of AEs, with the most common side effects reported as constipation and nausea. The analgesic effects of opioids were found to be effective and were gradually used in recent years for bursts of pain in patients with PHN (Portenoy et al., 1990; Arnér and Meyerson, 1988; Watson and Babul, 1998; Jadad et al., 1992). However, some elderly patients might not be able to tolerate the side effects of opioids, and caution should be exercised when using opioids.

The DIC values calculated for both consistency and inconsistency models were demonstrated to exhibit proximity, indicating robust agreement in model fit across the NMA framework (Ades et al., 2006). Heterogeneity tests were performed and found that the NMA for AE incidence indicated significant heterogeneity of interventions between groups, which was greatly reduced by network regressivity analysis with RoB as a covariate, and this indicated that the quality of the literature had a significant effect on heterogeneity. Publication bias and small-sample effects for clinical effectiveness rates were assessed using comparison-adjusted funnel plots. A statistically significant risk of publication bias or small-sample effect (P < 0.01) for clinical effectiveness rates was determined by the Egger regression test, which suggested that the reliability of the combined estimates may be compromised. Egger regression tests showed that asymmetry in clinical effectiveness rates might have an impact on pain intensity outcomes: (1) distortion of effect sizes and overestimation of pain relief: selective publication of positive results might lead to exaggerated drug efficacy, whereas the absence of negative results might lead to distorted safety assessments. (2) Decreased reliability of efficacy ranking: Publication bias caused an uneven evidence base for different drugs, which led to lower SUCRA rankings. However, publication bias also had an impact on the efficacy assessment of PHN drug treatment, so it was recommended to optimize the strategy of drug treatment in the clinic.

Ultimately, it was anticipated that the direct and indirect evidence synthesized through this investigation would be systematically leveraged to evaluate the comparative efficacy and safety of various drug interventions for PHN, thereby enabling the formulation of evidence-based therapeutic strategies to optimize pain management protocols for this neurological condition.

## 5 Limitations

There were some limitations in this study. Firstly, there were flaws in the study design, and a portion of the included RCTs had a high risk of bias in quality assessment, which might lead to an increase in the heterogeneity of the study results. Secondly, the Egger test indicated a publication bias or a small sample effect (P < 0.01) in the clinical effective rate, which might lead to unreliable results. Third, due to the limited amount of literature included in this study, certain interventions had a low number of cases in a certain efficacy indicator, which biased the results of the study. In conclusion, to be able to provide more scientific evidence, more high-quality RCTs are needed for further analysis in the future.

## 6 Conclusion

For pain management in PHN, capsaicin patches showed a statistically significant intervention on pain intensity, and hydromorphone was significant in improving sleep quality and reducing pain questionnaire scores. However, calcium channel modulators (pregabalin, gabapentin) were more relevant in the clinical management of PHN patients in terms of comprehensive treatment as well as improvement in quality of life.

## Data Availability

The original contributions presented in the study are included in the article/Supplementary Material, further inquiries can be directed to the corresponding author.
