# Supplementary material for: Efficacy and safety of different medications compared for the treatment of postherpetic neuralgia: a network meta-analysis
Source: Front Pharmacol. 2025 Jul 30;16:1614587. doi: 10.3389/fphar.2025.1614587 (PMC12343574; doi:10.3389/fphar.2025.1614587)
Supplement: Supplementary file 1 [file DataSheet2.pdf]

Trace and density plots

A.

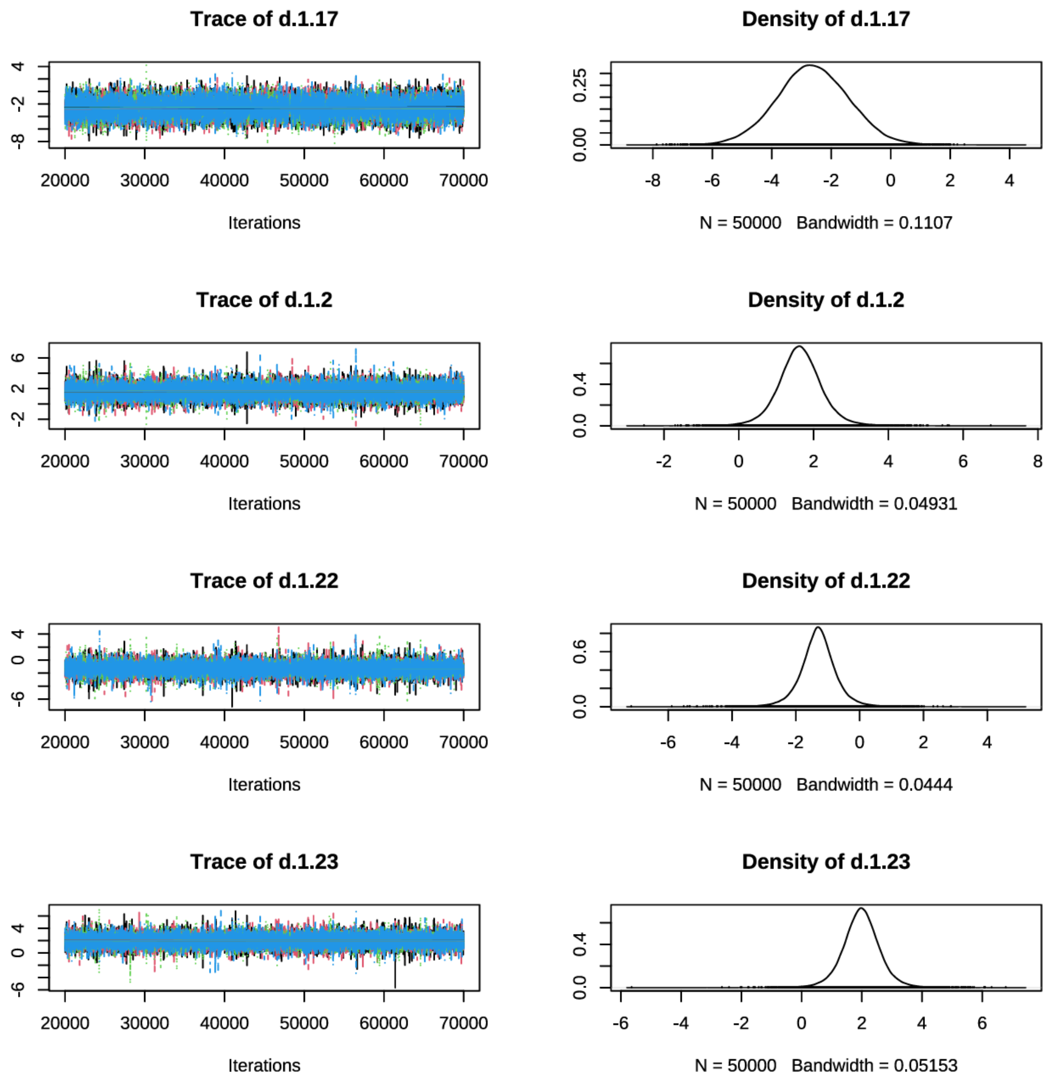

1=Pregabalin,2=Placebo,17=5% lidocaine medicated plasters,

22=hydromorphone,23=Routine drug treatment.

B.

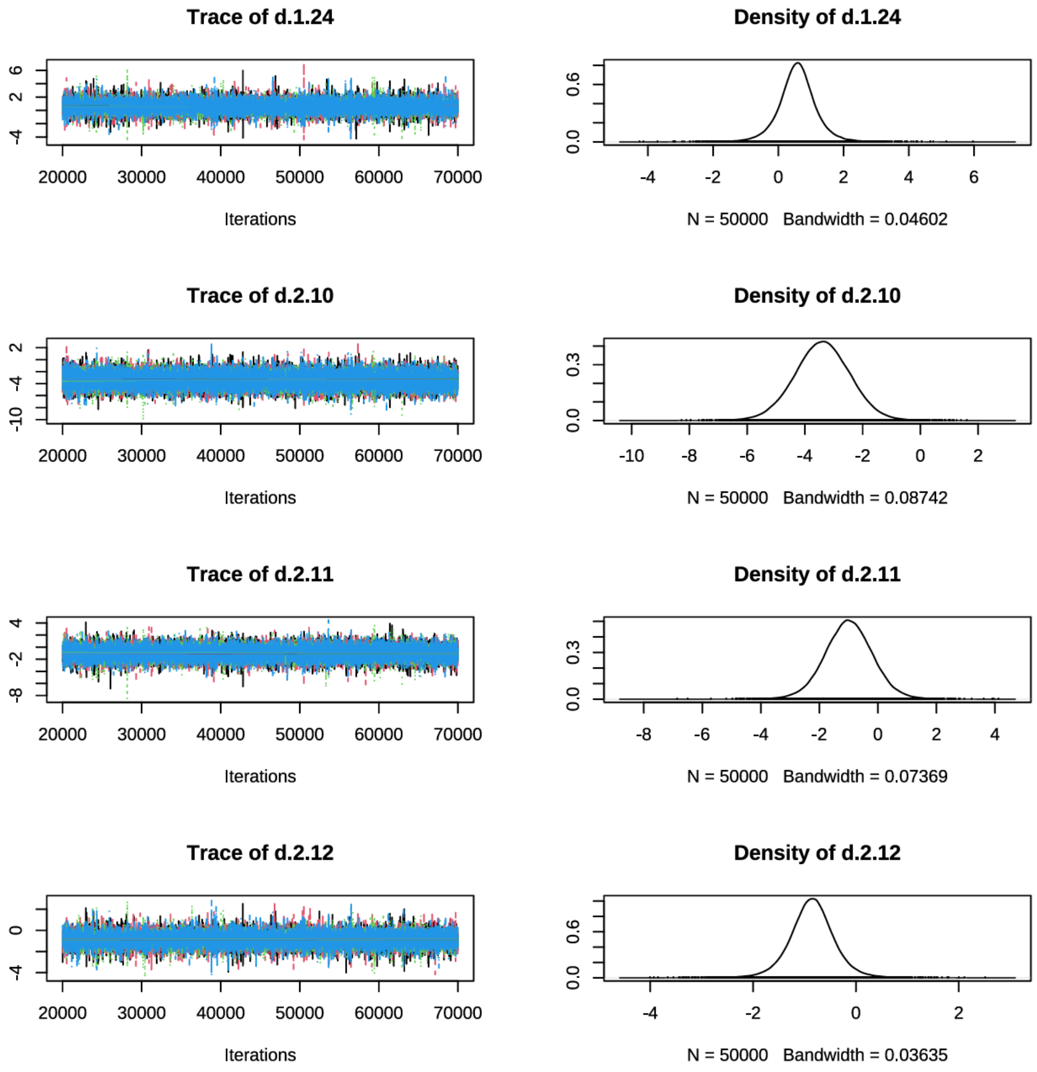

1=Pregabalin,2=Placebo, 10=8% lidocaine,11=TCA,12=Gabapentin Enacarbil 1200mg, 24=Nortriptyline.

C.

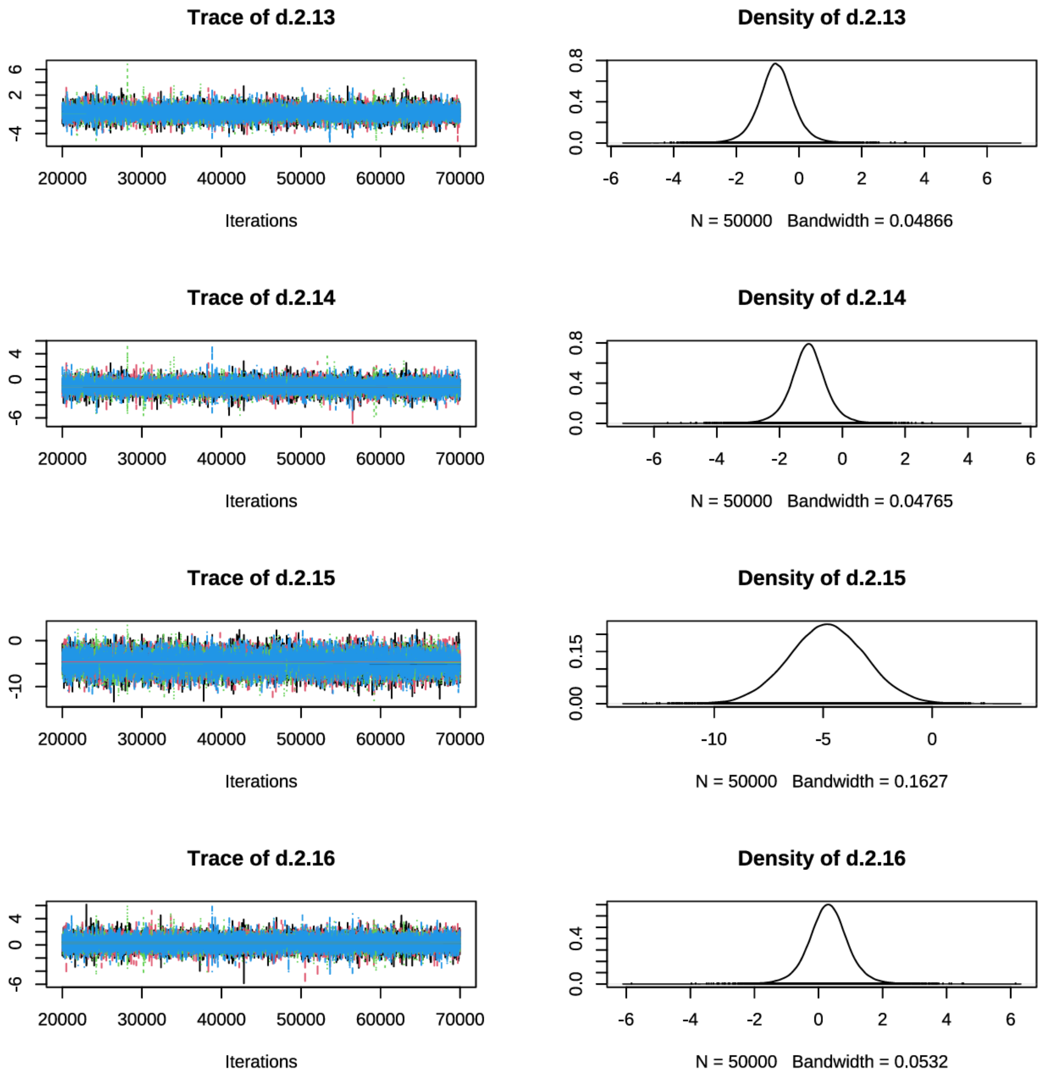

2=Placebo, 13=Gabapentin Enacarbil 2400mg,14=Gabapentin Enacarbil 3600mg,15=Gastroretentive Gabapentin,16=Gabapentin 1800mg.

D.

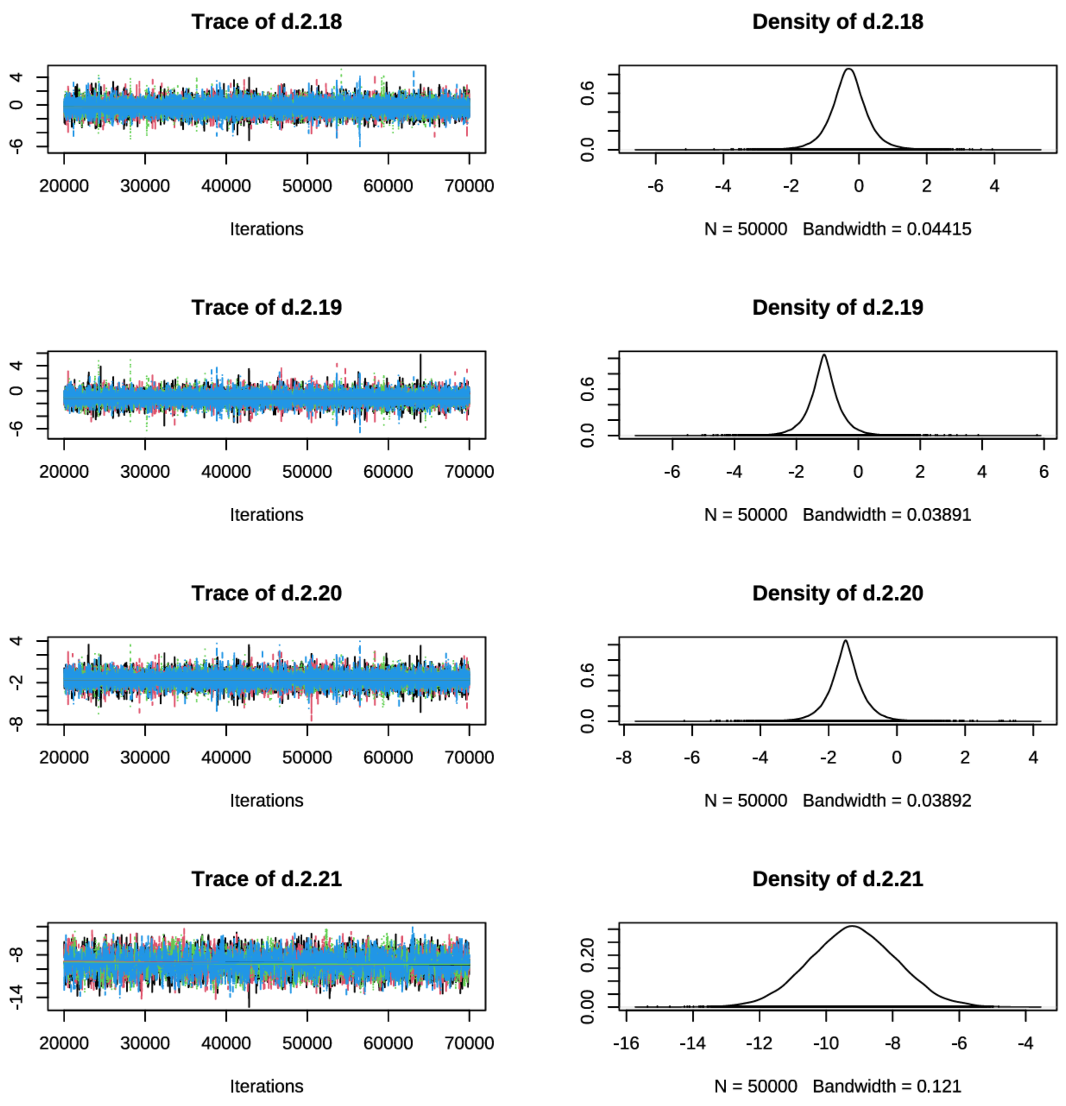

2=Placebo, 18=Tramadol 400mg,19 =Crisugabalin 40mg,20=Crisugabalin 80mg,21=NGX-4010 8% capsaicin patch.

E.

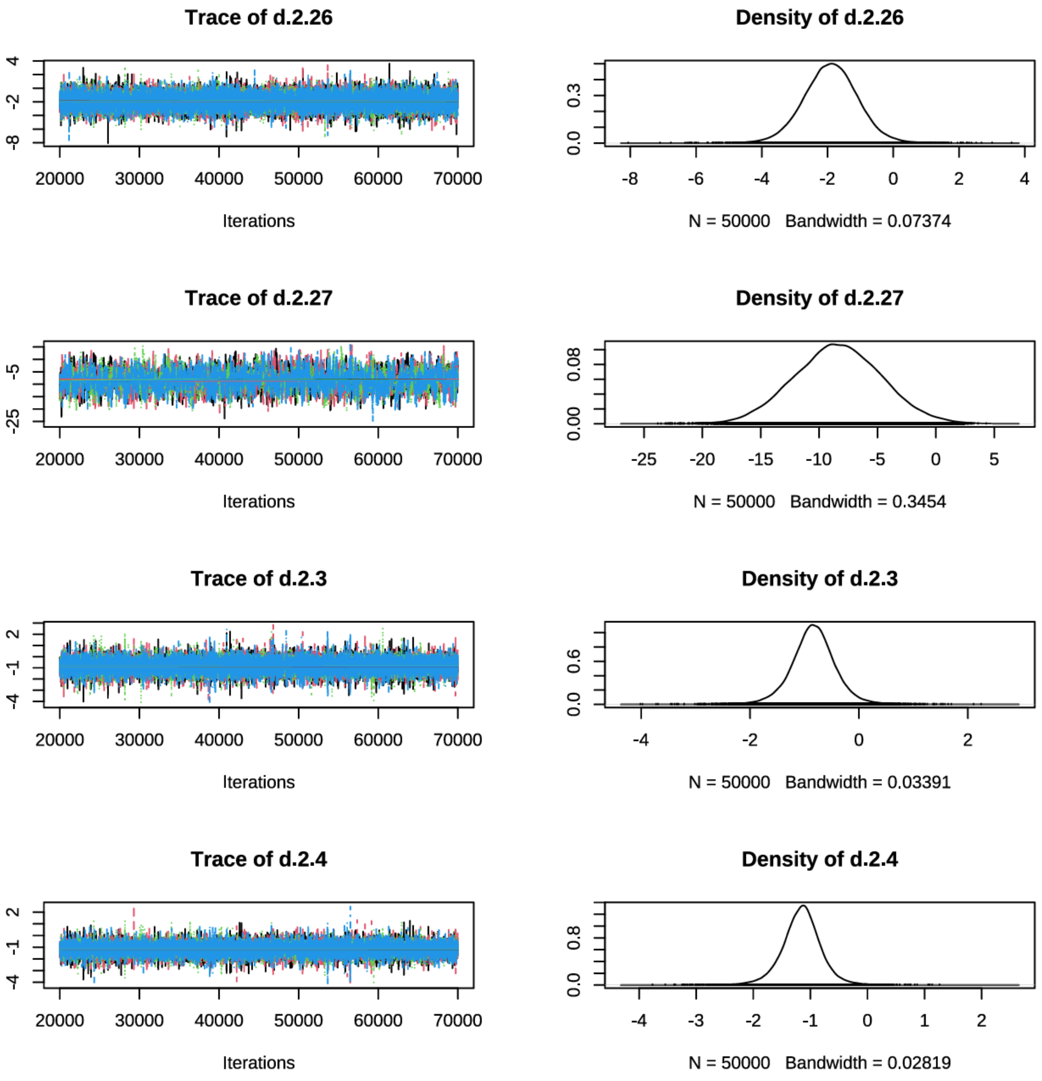

2=Placebo,3=Pregabalin150mg,4=Pregabalin300mg,26=Opioids,27=Tramadol 100mg.

F.

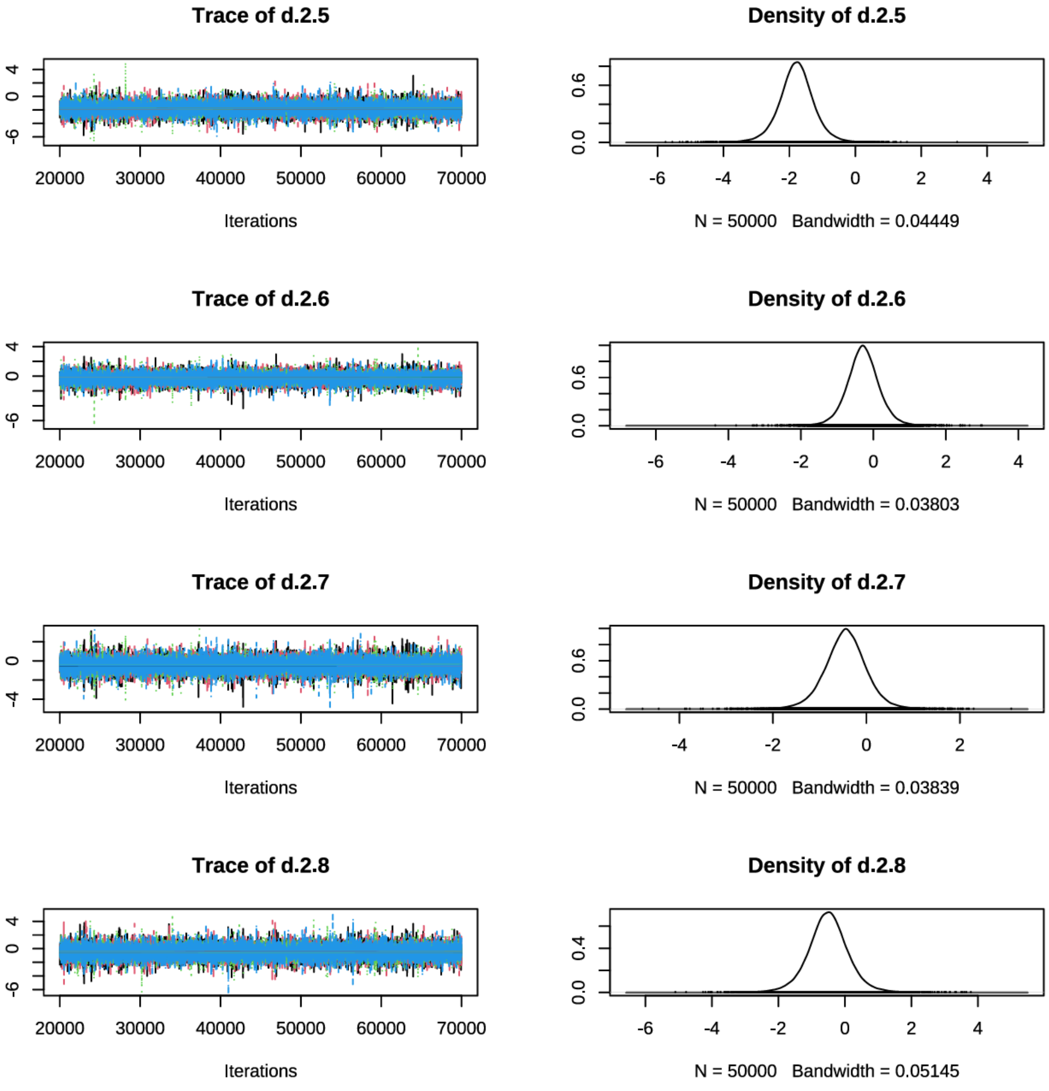

2=Placebo, 5=Pregabalin600mg,6=Gabapentin ER QD,7=Gabapentin ER BID,8=GW406381 25mg.

G.

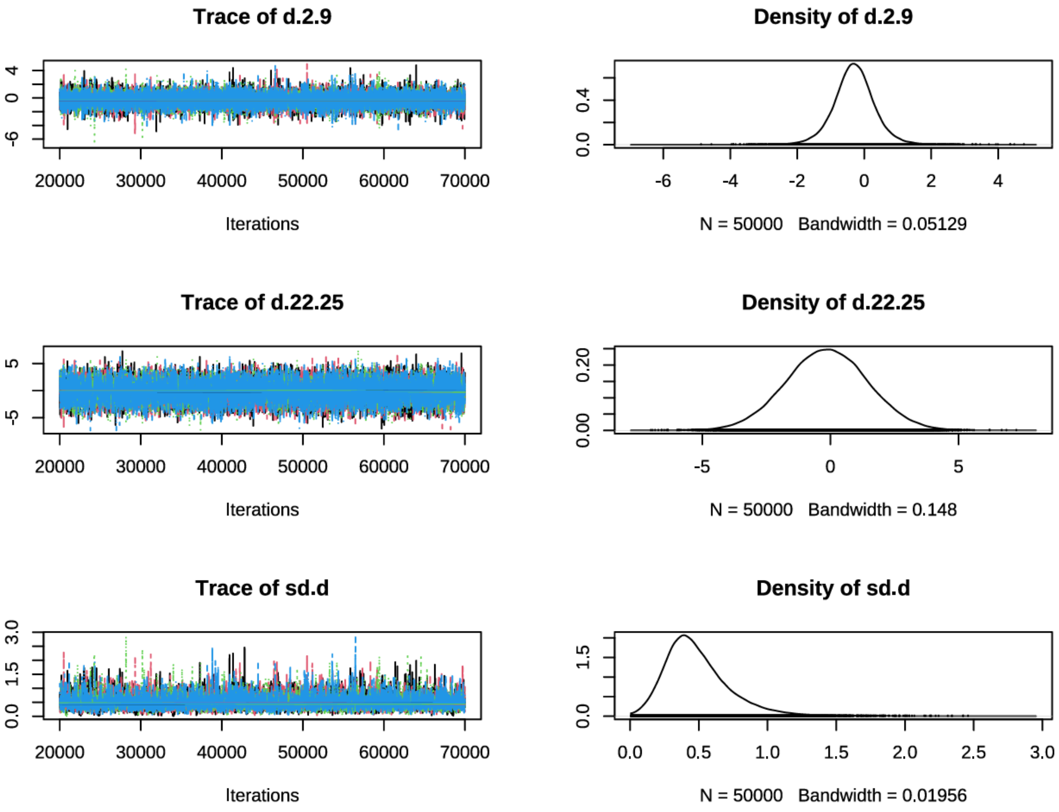

2=Placebo,9=GW406381 30mg, 22=hydromorphone,

25=morphine.sd=standard deviation
