# Supplementary material for: Efficacy and safety of different medications compared for the treatment of postherpetic neuralgia: a network meta-analysis
Source: Front Pharmacol. 2025 Jul 30;16:1614587. doi: 10.3389/fphar.2025.1614587 (PMC12343574; doi:10.3389/fphar.2025.1614587)
Supplement: Supplementary file 2 [file DataSheet4.pdf]

The Results of a network meta-analysis comparing pairwise

**1**																										
**-1.63 (-2.87, -0.44)**	**2**																									
-0.79 (-2.29, 0.67)	0.83 (-0.01, 1.7)	**3**																								
-0.47 (-1.9, 0.94)	1.15 (0.45, 1.89)	0.32 (-0.52, 1.18)	**4**																							
0.15 (-1.52, 1.8)	1.78 (0.67, 2.91)	0.95 (-0.21, 2.1)	0.63 (-0.51, 1.74)	**5**																						
-1.33 (-2.9, 0.16)	0.29 (-0.65, 1.22)	-0.54 (-1.82, 0.71)	-0.86 (-2.07, 0.29)	**-1.49 (-2.96, -0.04)**	**6**																					
-1.18 (-2.72, 0.35)	0.45 (-0.48, 1.4)	-0.39 (-1.65, 0.89)	-0.71 (-1.89, 0.47)	-1.33 (-2.8, 0.14)	0.15 (-0.76, 1.11)	**7**																				
-1.12 (-2.91, 0.63)	0.5 (-0.79, 1.79)	-0.33 (-1.88, 1.21)	-0.65 (-2.15, 0.81)	-1.28 (-3, 0.43)	0.21 (-1.38, 1.82)	0.05 (-1.56, 1.64)	**8**																			
-1.33 (-3.12, 0.42)	0.3 (-0.99, 1.59)	-0.53 (-2.1, 1.01)	-0.85 (-2.35, 0.6)	-1.48 (-3.2, 0.22)	0.01 (-1.58, 1.61)	-0.15 (-1.76, 1.44)	-0.2 (-1.49, 1.08)	**9**																		
1.78 (-0.49, 4.02)	3.41 (1.51, 5.31)	2.57 (0.49, 4.65)	2.26 (0.21, 4.26)	1.63 (-0.6, 3.8)	3.12 (1, 5.24)	2.96 (0.83, 5.07)	2.91 (0.6, 5.2)	3.11 (0.81, 5.39)	**10**																	
-0.63 (-2.7, 1.39)	0.99 (-0.65, 2.64)	0.16 (-1.7, 2.01)	-0.16 (-1.96, 1.62)	-0.79 (-2.78, 1.21)	0.7 (-1.17, 2.61)	0.55 (-1.34, 2.44)	0.5 (-1.6, 2.6)	0.7 (-1.4, 2.79)	-2.41 (-4.93, 0.11)	**11**																
-0.77 (-2.3, 0.73)	0.86 (-0.05, 1.77)	0.02 (-1.23, 1.26)	-0.3 (-1.47, 0.84)	-0.93 (-2.37, 0.52)	0.56 (-0.72, 1.88)	0.41 (-0.91, 1.7)	0.35 (-1.22, 1.94)	0.55 (-1.02, 2.14)	**-2.55 (-4.64, -0.44)**	-0.14 (-2.02, 1.74)	**12**															
-0.91 (-2.64, 0.8)	0.72 (-0.5, 1.94)	-0.12 (-1.6, 1.37)	-0.44 (-1.87, 0.96)	-1.06 (-2.72, 0.6)	0.43 (-1.09, 1.97)	0.27 (-1.27, 1.8)	0.22 (-1.55, 2)	0.42 (-1.35, 2.2)	**-2.69 (-4.94, -0.42)**	-0.28 (-2.32, 1.77)	-0.14 (-1.35, 1.08)	**13**														
-0.55 (-2.27, 1.14)	1.08 (-0.12, 2.29)	0.25 (-1.24, 1.7)	-0.07 (-1.49, 1.31)	-0.7 (-2.35, 0.94)	0.79 (-0.72, 2.33)	0.64 (-0.91, 2.14)	0.58 (-1.19, 2.35)	0.78 (-0.98, 2.55)	**-2.33 (-4.55, -0.07)**	0.08 (-1.95, 2.11)	0.23 (-0.98, 1.42)	0.36 (-0.94, 1.66)	**14**													
3.18 (-0.55, 6.83)	4.81 (1.3, 8.25)	3.97 (0.38, 7.52)	3.65 (0.07, 7.17)	3.02 (-0.65, 6.65)	4.52 (0.91, 8.09)	4.36 (0.74, 7.93)	4.31 (0.58, 7.99)	4.51 (0.78, 8.19)	1.41 (-2.61, 5.35)	3.82 (-0.08, 7.63)	3.95 (0.32, 7.51)	4.1 (0.37, 7.74)	3.73 (0.01, 7.38)	**15**												
**-1.93 (-3.73, -0.18)**	-0.3 (-1.6, 1)	-1.13 (-2.71, 0.41)	-1.45 (-2.96, 0.01)	**-2.08 (-3.81, -0.36)**	-0.59 (-2.18, 1.01)	-0.75 (-2.37, 0.84)	-0.8 (-2.64, 1.02)	-0.6 (-2.43, 1.23)	**-3.71 (-5.99, -1.4)**	-1.29 (-3.39, 0.79)	-1.15 (-2.75, 0.42)	-1.02 (-2.81, 0.76)	-1.38 (-3.16, 0.38)	**-5.11 (-8.79, -1.37)**	**16**											
2.63 (0.21, 5.01)	4.26 (1.81, 6.66)	3.42 (0.84, 5.96)	3.1 (0.54, 5.6)	2.48 (-0.21, 5.12)	3.97 (1.34, 6.56)	3.81 (1.18, 6.4)	3.75 (0.99, 6.48)	3.96 (1.19, 6.68)	0.85 (-2.23, 3.93)	3.25 (0.3, 6.17)	3.4 (0.79, 5.97)	3.54 (0.8, 6.24)	3.18 (0.46, 5.86)	-0.56 (-4.81, 3.75)	4.56 (1.78, 7.29)	**17**										
-1.33 (-3.05, 0.36)	0.3 (-0.89, 1.49)	-0.53 (-2.01, 0.93)	-0.85 (-2.27, 0.52)	-1.48 (-3.14, 0.16)	0.01 (-1.5, 1.54)	-0.15 (-1.67, 1.35)	-0.2 (-1.97, 1.55)	0 (-1.76, 1.76)	**-3.11 (-5.33, -0.87)**	-0.7 (-2.71, 1.32)	-0.56 (-2.06, 0.94)	-0.42 (-2.13, 1.29)	-0.78 (-2.49, 0.92)	**-4.5 (-8.15, -0.83)**	0.6 (-1.16, 2.37)	**-3.96 (-6.64, -1.26)**	**18**									
-0.53 (-2.22, 1.12)	1.1 (-0.04, 2.25)	0.26 (-1.17, 1.69)	-0.05 (-1.42, 1.28)	-0.68 (-2.29, 0.92)	0.81 (-0.66, 2.3)	0.65 (-0.84, 2.13)	0.6 (-1.11, 2.32)	0.8 (-0.92, 2.53)	**-2.31 (-4.5, -0.09)**	0.11 (-1.89, 2.11)	0.24 (-1.22, 1.71)	0.38 (-1.3, 2.04)	0.02 (-1.64, 1.67)	**-3.71 (-7.32, -0.03)**	1.4 (-0.32, 3.12)	**-3.16 (-5.82, -0.48)**	0.8 (-0.86, 2.46)	**19**								
-0.13 (-1.82, 1.52)	1.5 (0.35, 2.65)	0.66 (-0.77, 2.09)	0.35 (-1.03, 1.69)	-0.28 (-1.9, 1.33)	1.2 (-0.26, 2.7)	1.05 (-0.45, 2.53)	1 (-0.73, 2.71)	1.2 (-0.52, 2.91)	-1.91 (-4.11, 0.3)	0.51 (-1.5, 2.5)	0.64 (-0.83, 2.1)	0.78 (-0.91, 2.44)	0.42 (-1.24, 2.08)	-3.31 (-6.93, 0.36)	1.8 (0.06, 3.53)	**-2.76 (-5.41, -0.08)**	1.2 (-0.46, 2.86)	0.4 (-0.75, 1.55)	**20**							
7.61 (4.76, 10.41)	9.24 (6.66, 11.78)	8.4 (5.7, 11.09)	8.08 (5.41, 10.73)	7.45 (4.64, 10.24)	8.95 (6.21, 11.68)	8.78 (6.05, 11.52)	8.74 (5.86, 11.57)	8.94 (6.07, 11.78)	5.83 (2.63, 9.01)	8.24 (5.18, 11.28)	8.38 (5.65, 11.09)	8.52 (5.67, 11.36)	8.16 (5.31, 10.99)	4.43 (0.12, 8.76)	9.53 (6.66, 12.41)	4.98 (1.49, 8.53)	8.94 (6.12, 11.75)	8.13 (5.32, 10.91)	7.74 (4.93, 10.51)	**21**						
1.3 (0.1, 2.5)	2.93 (1.24, 4.67)	2.09 (0.2, 4.02)	1.78 (-0.08, 3.62)	1.15 (-0.89, 3.21)	2.63 (0.73, 4.63)	2.48 (0.54, 4.45)	2.43 (0.3, 4.6)	2.63 (0.51, 4.81)	-0.48 (-3.01, 2.08)	1.93 (-0.41, 4.3)	2.07 (0.15, 4.01)	2.21 (0.12, 4.32)	1.85 (-0.23, 3.96)	-1.88 (-5.72, 2.03)	3.23 (1.1, 5.4)	-1.33 (-3.97, 1.36)	2.63 (0.56, 4.73)	1.83 (-0.21, 3.91)	1.43 (-0.62, 3.52)	**-6.31 (-9.36, -3.2)**	**22**					
**-1.98 (-3.26, -0.7)**	-0.35 (-2.09, 1.45)	-1.19 (-3.13, 0.79)	-1.5 (-3.41, 0.4)	**-2.13 (-4.23, -0.02)**	-0.64 (-2.6, 1.39)	-0.8 (-2.8, 1.21)	-0.85 (-3.02, 1.35)	-0.66 (-2.82, 1.57)	**-3.76 (-6.32, -1.15)**	-1.35 (-3.74, 1.08)	-1.21 (-3.18, 0.8)	-1.07 (-3.21, 1.09)	-1.43 (-3.55, 0.72)	**-5.15 (-9.01, -1.22)**	-0.05 (-2.21, 2.18)	**-4.6 (-7.32, -1.89)**	-0.65 (-2.75, 1.51)	-1.45 (-3.54, 0.68)	-1.85 (-3.94, 0.29)	**-9.59 (-12.66, -**	**-3.28 (-5.05, -1.52)**	**23**				
-0.58 (-1.8, 0.64)	1.05 (-0.66, 2.8)	0.21 (-1.7, 2.15)	-0.11 (-1.98, 1.76)	-0.74 (-2.77, 1.34)	0.75 (-1.17, 2.75)	0.6 (-1.36, 2.59)	0.54 (-1.59, 2.72)	0.75 (-1.38, 2.92)	-2.36 (-4.9, 0.22)	0.05 (-2.31, 2.45)	0.19 (-1.75, 2.16)	0.33 (-1.78, 2.45)	-0.04 (-2.11, 2.09)	-3.76 (-7.6, 0.16)	1.35 (-0.79, 3.53)	**-3.21 (-5.86, -0.52)**	0.75 (-1.33, 2.86)	-0.05 (-2.11, 2.05)	-0.45 (-2.51, 1.64)	**-8.2 (-11.23, -**	**-1.88 (-3.6, -0.16)**	1.4 (-0.37, 3.16)	**24**			
1.4 (-1.95, 4.75)	3.04 (-0.52, 6.58)	2.2 (-1.44, 5.83)	1.88 (-1.74, 5.48)	1.26 (-2.47, 4.97)	2.74 (-0.91, 6.43)	2.58 (-1.11, 6.24)	2.54 (-1.26, 6.31)	2.73 (-1.05, 6.49)	-0.37 (-4.41, 3.62)	2.03 (-1.89, 5.96)	2.18 (-1.48, 5.83)	2.32 (-1.42, 6.07)	1.95 (-1.77, 5.7)	-1.76 (-6.75, 3.17)	3.34 (-0.45, 7.12)	-1.21 (-5.32, 2.87)	2.74 (-1, 6.48)	1.94 (-1.78, 5.65)	1.54 (-2.19, 5.26)	**-6.21 (-10.53, -1.8)**	0.1 (-3.05, 3.23)	3.38 (-0.21, 6.96)	1.99 (-1.59, 5.54)	**25**		
0.26 (-1.79, 2.3)	1.89 (0.25, 3.56)	1.06 (-0.8, 2.93)	0.74 (-1.06, 2.53)	0.11 (-1.87, 2.12)	1.6 (-0.27, 3.51)	1.45 (-0.46, 3.35)	1.39 (-0.7, 3.5)	1.59 (-0.5, 3.7)	-1.51 (-4.02, 1.01)	0.89 (-0.79, 2.6)	1.04 (-0.84, 2.94)	1.17 (-0.87, 3.23)	0.81 (-1.22, 2.86)	-2.92 (-6.75, 0.96)	2.19 (0.1, 4.31)	-2.36 (-5.27, 0.57)	1.6 (-0.44, 3.63)	0.79 (-1.2, 2.8)	0.4 (-1.6, 2.4)	**-7.34 (-10.4, -4.27)**	-1.03 (-3.42, 1.32)	2.24 (-0.17, 4.65)	0.85 (-1.55, 3.22)	-1.14 (-5.06, 2.8)	**26**	
6.85 (-0.8, 14.39)	8.48 (0.94, 15.88)	7.65 (0.04, 15.1)	7.32 (-0.26, 14.77)	6.7 (-0.95, 14.2)	8.19 (0.6, 15.66)	8.03 (0.42, 15.51)	7.99 (0.32, 15.52)	8.18 (0.52, 15.72)	5.07 (-2.67, 12.74)	7.47 (-0.28, 15.06)	7.63 (0.01, 15.1)	7.76 (0.1, 15.28)	7.4 (-0.26, 14.91)	3.64 (-4.69, 12.09)	8.78 (1.15, 16.3)	4.22 (-3.67, 12.02)	8.18 (0.56, 15.68)	7.38 (-0.25, 14.88)	6.98 (-0.64, 14.47)	-0.75 (-8.77, 7.15)	5.54 (-2.2, 13.16)	8.83 (1.07, 16.46)	7.42 (-0.33, 15.05)	5.42 (-2.81, 13.66)	6.58 (-1.16, 14.15)	**27**

A. The network meta-analysis results of pain scores comparisons.

Note: Comparisons between treatments should be read from left to right, and the estimate is in the cell in common between the column-defining treatment and the row-defining treatment. The lower the efficacy value after treatment, the more significant the efficacy of the column. Significant results are set in boldface.

**1**																															
1.36 (0.61, 2.06)	**2**																														
0.19 (-0.83, 1.08)	**-1.17 (-1.82, -0.58)**	**3**																													
0.2 (-0.72, 1.05)	**-1.15 (-1.68, -0.67)**	**0.02 (-0.54, 0.6)**	**4**																												
**-0.39 (-1.5, 0.6)**	**-1.75 (-2.55, -1.03)**	**-0.58 (-1.31, 0.15)**	**-0.6 (-1.3, 0.1)**	**5**																											
0.92 (-0.11, 1.92)	**-0.43 (-1.14, 0.28)**	0.74 (-0.16, 1.71)	0.71 (-0.13, 1.61)	1.31 (0.33, 2.38)	**6**																										
1.03 (0, 2.04)	**-0.32 (-1.03, 0.4)**	0.85 (-0.05, 1.84)	0.83 (-0.01, 1.73)	1.43 (0.42, 2.51)	**0.11 (-0.57, 0.81)**	**7**																									
0.42 (-1.26, 1.87)	**-0.95 (-2.39, 0.37)**	0.22 (-1.36, 1.71)	0.2 (-1.34, 1.62)	0.81 (-0.87, 2.33)	**-0.52 (-2.11, 0.97)**	**-0.63 (-2.22, 0.85)**	**8**																								
0.16 (-1.54, 1.59)	**-1.19 (-2.63, 0.05)**	-0.03 (-1.6, 1.41)	-0.05 (-1.59, 1.32)	0.56 (-1.1, 2.04)	**-0.77 (-2.35, 0.65)**	**-0.88 (-2.47, 0.54)**	**-0.25 (-1.29, 0.8)**	**9**																							
0.18 (-1.68, 1.9)	**-1.18 (-2.91, 0.38)**	0 (-1.82, 1.72)	-0.01 (-1.82, 1.61)	0.58 (-1.29, 2.34)	**-0.75 (-2.62, 0.96)**	**-0.86 (-2.72, 0.87)**	-0.21 (-2.43, 1.86)	0.04 (-2.14, 2.11)	**10**																						
0.62 (-0.35, 1.55)	**-0.75 (-1.36, -0.11)**	0.43 (-0.42, 1.35)	0.4 (-0.39, 1.25)	1 (0.07, 2.04)	**-0.31 (-1.26, 0.64)**	**-0.42 (-1.37, 0.53)**	0.21 (-1.23, 1.78)	0.45 (-0.94, 2.01)	0.42 (-1.26, 2.29)	**11**																					
0.88 (-0.21, 1.91)	**-0.48 (-1.27, 0.32)**	0.69 (-0.3, 1.73)	0.67 (-0.27, 1.63)	1.27 (0.21, 2.39)	-0.05 (-1.12, 1)	**-0.16 (-1.23, 0.93)**	0.46 (-1.05, 2.11)	0.72 (-0.76, 2.34)	0.7 (-1.06, 2.62)	0.27 (-0.49, 1.01)	**12**																				
0.51 (-0.55, 1.53)	**-0.85 (-1.59, -0.08)**	0.32 (-0.6, 1.34)	0.3 (-0.57, 1.24)	0.9 (-0.12, 2.02)	**-0.41 (-1.45, 0.61)**	**-0.53 (-1.56, 0.51)**	0.1 (-1.39, 1.72)	0.35 (-1.11, 1.95)	0.33 (-1.41, 2.24)	**-0.1 (-0.84, 0.62)**	**-0.37 (-1.16, 0.43)**	**13**																			
0.4 (-0.67, 1.48)	**-0.96 (-1.73, -0.13)**	0.22 (-0.76, 1.3)	0.19 (-0.71, 1.2)	0.79 (-0.26, 1.98)	**-0.52 (-1.55, 0.58)**	**-0.64 (-1.67, 0.46)**	0 (-1.52, 1.69)	0.23 (-1.22, 1.93)	0.22 (-1.51, 2.14)	**-0.2 (-1.2, 0.82)**	**-0.48 (-1.57, 0.68)**	-0.11 (-1.19, 1.01)	**14**																		
0.62 (-0.37, 1.74)	**-0.74 (-1.46, 0.17)**	0.44 (-0.49, 1.62)	0.42 (-0.44, 1.49)	1.02 (0.02, 2.27)	**-0.3 (-1.27, 0.86)**	**-0.42 (-1.4, 0.78)**	0.23 (-1.29, 1.97)	0.47 (-0.98, 2.2)	0.45 (-1.23, 2.38)	0.02 (-0.92, 1.09)	**-0.25 (-1.29, 0.95)**	0.12 (-0.92, 1.28)	0.22 (-0.47, 1.03)	**15**																	
**0.06 (-0.89, 0.89)**	**-1.29 (-2.06, -0.6)**	**-0.12 (-1.08, 0.83)**	**-0.14 (-1.04, 0.72)**	0.45 (-0.59, 1.53)	**-0.85 (-1.91, 0.1)**	**-0.97 (-2.04, -0.01)**	-0.34 (-1.86, 1.25)	-0.09 (-1.56, 1.49)	-0.12 (-1.88, 1.73)	**-0.54 (-1.55, 0.36)**	**-0.81 (-1.91, 0.22)**	**-0.44 (-1.54, 0.57)**	**-0.33 (-1.48, 0.69)**	**-0.56 (-1.81, 0.44)**	**16**																
0.12 (-1.02, 1.22)	**-1.23 (-2.11, -0.38)**	-0.06 (-1.1, 1.03)	**-0.08 (-1.09, 0.93)**	0.51 (-0.61, 1.69)	**-0.81 (-1.93, 0.31)**	**-0.92 (-2.04, 0.2)**	-0.29 (-1.83, 1.39)	-0.05 (-1.54, 1.61)	-0.05 (-1.86, 1.86)	**-0.49 (-1.57, 0.55)**	**-0.76 (-1.93, 0.42)**	**-0.38 (-1.55, 0.74)**	**-0.28 (-1.5, 0.88)**	**-0.5 (-1.76, 0.6)**	0.06 (-1.06, 1.21)	**17**															
0.48 (-0.56, 1.52)	**-0.87 (-1.61, -0.11)**	0.3 (-0.63, 1.31)	0.28 (-0.6, 1.2)	0.88 (-0.15, 1.99)	**-0.44 (-1.46, 0.6)**	**-0.55 (-1.57, 0.5)**	0.08 (-1.4, 1.7)	0.33 (-1.1, 1.94)	0.31 (-1.46, 2.2)	**-0.12 (-1.1, 0.85)**	**-0.38 (-1.5, 0.7)**	-0.02 (-1.08, 1.04)	0.09 (-1.04, 1.16)	**-0.13 (-1.35, 0.88)**	0.42 (-0.57, 1.52)	0.36 (-0.76, 1.52)	**18**														
0.45 (-0.6, 1.48)	**-0.9 (-1.65, -0.15)**	0.27 (-0.67, 1.27)	0.24 (-0.65, 1.16)	0.84 (-0.19, 1.96)	**-0.47 (-1.49, 0.55)**	**-0.58 (-1.62, 0.44)**	0.05 (-1.45, 1.65)	0.3 (-1.13, 1.89)	0.28 (-1.47, 2.15)	**-0.16 (-1.14, 0.81)**	**-0.42 (-1.51, 0.68)**	-0.06 (-1.11, 1.01)	0.05 (-1.08, 1.12)	**-0.18 (-1.37, 0.84)**	0.38 (-0.6, 1.47)	0.33 (-0.8, 1.48)	**-0.04 (-0.74, 0.68)**	**19**													
0.86 (0.03, 1.6)	**-0.49 (-0.82, -0.23)**	0.67 (0, 1.37)	0.65 (0.07, 1.23)	1.25 (0.47, 2.08)	**-0.06 (-0.85, 0.68)**	**-0.18 (-0.98, 0.57)**	0.45 (-0.91, 1.92)	0.69 (-0.59, 2.15)	0.67 (-0.91, 2.42)	**0.25 (-0.48, 0.92)**	**-0.02 (-0.87, 0.81)**	0.35 (-0.48, 1.13)	0.46 (-0.45, 1.27)	0.24 (-0.74, 1.01)	0.79 (0.05, 1.59)	0.73 (-0.19, 1.65)	0.37 (-0.46, 1.15)	0.41 (-0.42, 1.18)	**20**												
0.84 (-0.15, 1.81)	**-0.51 (-1.16, 0.16)**	0.66 (-0.2, 1.62)	0.64 (-0.16, 1.51)	1.24 (0.29, 2.31)	**-0.08 (-1.04, 0.89)**	**-0.19 (-1.16, 0.79)**	0.45 (-1.02, 2.04)	0.7 (-0.73, 2.26)	0.67 (-1, 2.51)	0.24 (-0.69, 1.13)	-0.03 (-1.06, 1)	0.34 (-0.68, 1.33)	0.46 (-0.62, 1.46)	0.23 (-0.9, 1.19)	0.78 (-0.17, 1.82)	0.73 (-0.35, 1.84)	0.36 (-0.63, 1.36)	0.4 (-0.58, 1.39)	**-0.01 (-0.71, 0.74)**	**21**											
2.34 (0.6, 4.15)	0.98 (-0.57, 2.66)	2.15 (0.78, 3.72)	2.13 (0.63, 3.79)	**2.74 (1.17, 4.43)**	1.41 (-0.29, 3.26)	1.3 (-0.43, 3.12)	1.96 (-0.12, 4.12)	2.2 (0.15, 4.34)	2.16 (-0.05, 4.57)	1.73 (0.04, 3.49)	1.47 (-0.32, 3.3)	1.82 (0.08, 3.63)	1.96 (0.14, 3.75)	1.72 (-0.13, 3.5)	2.28 (0.55, 4.12)	2.22 (0.39, 4.08)	1.85 (0.1, 3.69)	1.89 (0.14, 3.74)	1.48 (-0.09, 3.19)	1.49 (-0.23, 3.29)	**22**										
2.99 (0.69, 6.74)	1.62 (-0.53, 5.35)	2.79 (0.73, 6.43)	2.77 (0.65, 6.49)	**3.38 (1.17, 7.11)**	2.06 (-0.22, 5.81)	1.95 (-0.33, 5.67)	2.62 (0.08, 6.45)	2.89 (0.3, 6.69)	2.85 (0.06, 6.89)	2.36 (0.13, 6.12)	2.11 (-0.19, 5.91)	2.47 (0.17, 6.24)	2.59 (0.26, 6.37)	2.35 (-0.02, 6.15)	2.92 (0.63, 6.74)	2.86 (0.53, 6.64)	2.49 (0.16, 6.29)	2.52 (0.22, 6.31)	2.12 (-0.03, 5.85)	2.13 (-0.14, 5.87)	0.63 (-1.88, 4.58)	**23**									
0.68 (-0.63, 1.97)	**-0.67 (-1.76, 0.4)**	0.51 (-0.74, 1.75)	0.49 (-0.7, 1.66)	1.08 (-0.22, 2.43)	-0.24 (-1.54, 1.07)	**-0.35 (-1.65, 0.93)**	0.28 (-1.42, 2.07)	0.53 (-1.09, 2.32)	0.51 (-1.41, 2.48)	0.08 (-1.2, 1.3)	-0.19 (-1.55, 1.13)	0.17 (-1.15, 1.49)	0.29 (-1.07, 1.58)	0.06 (-1.39, 1.32)	0.62 (-0.66, 1.94)	0.56 (-0.81, 1.96)	0.2 (-1.11, 1.5)	0.23 (-1.08, 1.54)	**-0.17 (-1.29, 0.94)**	-0.17 (-1.42, 1.1)	**-1.65 (-3.66, 0.25)**	**-2.31 (-6.09, 0.16)**	**24**								
-0.07 (-2, 1.69)	**-1.41 (-3.22, 0.2)**	-0.24 (-2.12, 1.49)	-0.27 (-2.09, 1.44)	0.34 (-1.57, 2.14)	**-0.98 (-2.91, 0.76)**	**-1.09 (-3.01, 0.65)**	-0.45 (-2.72, 1.68)	-0.21 (-2.43, 1.93)	-0.25 (-1.61, 1.11)	-0.67 (-2.6, 1.07)	**-0.94 (-2.9, 0.86)**	-0.56 (-2.55, 1.23)	-0.46 (-2.44, 1.36)	-0.68 (-2.71, 1.07)	-0.11 (-2.06, 1.62)	-0.17 (-2.17, 1.64)	-0.55 (-2.49, 1.22)	-0.51 (-2.44, 1.26)	**-0.92 (-2.73, 0.72)**	**-0.92 (-2.82, 0.82)**	**-2.42 (-4.82, -0.12)**	**-3.09 (-7.18, -0.28)**	-0.75 (-2.77, 1.16)	**25**							
**-0.98 (-2.61, 0.61)**	**-2.33 (-4.12, -0.56)**	**-1.16 (-3.04, 0.73)**	**-1.17 (-3.03, 0.69)**	-0.59 (-2.49, 1.36)	**-1.9 (-3.82, 0.01)**	**-2.01 (-3.92, -0.11)**	**-1.39 (-3.61, 0.94)**	-1.15 (-3.38, 1.2)	-1.16 (-3.55, 1.35)	**-1.6 (-3.48, 0.27)**	**-1.87 (-3.81, 0.1)**	**-1.49 (-3.44, 0.42)**	-1.39 (-3.34, 0.54)	**-1.61 (-3.58, 0.28)**	**-1.05 (-2.87, 0.8)**	**-1.1 (-3.09, 0.88)**	**-1.46 (-3.43, 0.45)**	-1.42 (-3.38, 0.48)	**-1.83 (-3.64, -0.02)**	**-1.82 (-3.77, 0.07)**	**-3.33 (-5.76, -0.99)**	**-4.03 (-8.01, -1.14)**	**-1.66 (-3.73, 0.4)**	-0.93 (-3.34, 1.59)	**26**						
0.83 (-1.77, 3.44)	-0.53 (-3.22, 2.19)	0.65 (-2.1, 3.5)	0.62 (-2.12, 3.4)	1.22 (-1.58, 4.06)	-0.12 (-2.88, 2.69)	-0.22 (-2.99, 2.66)	0.41 (-2.57, 3.5)	0.66 (-2.33, 3.77)	0.65 (-2.49, 3.93)	0.22 (-2.59, 3.01)	-0.05 (-2.88, 2.8)	0.32 (-2.53, 3.16)	0.42 (-2.39, 3.21)	0.2 (-2.64, 2.96)	0.74 (-1.96, 3.59)	0.69 (-2.1, 3.57)	0.33 (-2.47, 3.23)	0.36 (-2.43, 3.19)	-0.03 (-2.73, 2.71)	-0.03 (-2.76, 2.77)	-1.53 (-4.68, 1.66)	-2.24 (-6.62, 1.4)	0.13 (-2.73, 3.16)	0.89 (-2.26, 4.21)	1.78 (-0.9, 4.63)	**27**					
0.65 (-0.72, 2.05)	**-0.7 (-2.22, 0.88)**	0.48 (-1.14, 2.2)	0.46 (-1.14, 2.12)	1.05 (-0.6, 2.81)	-0.27 (-1.95, 1.46)	-0.38 (-2.05, 1.34)	0.25 (-1.72, 2.4)	0.5 (-1.46, 2.63)	0.46 (-1.69, 2.85)	0.03 (-1.61, 1.74)	-0.22 (-1.94, 1.54)	0.14 (-1.54, 1.88)	0.26 (-1.47, 1.98)	0.03 (-1.74, 1.74)	0.6 (-1.01, 2.28)	0.53 (-1.22, 2.37)	0.16 (-1.54, 1.91)	0.2 (-1.5, 1.95)	-0.2 (-1.75, 1.4)	-0.19 (-1.88, 1.52)	**-1.69 (-3.9, 0.55)**	**-2.38 (-6.24, 0.42)**	-0.02 (-1.92, 1.94)	0.72 (-1.48, 3.07)	1.65 (-0.45, 3.78)	-0.17 (-3.08, 2.77)	**28**				
2.18 (0.37, 4.36)	0.83 (-1.07, 3.14)	2.02 (0.03, 4.41)	1.99 (0.04, 4.36)	2.59 (0.55, 4.99)	1.26 (-0.75, 3.68)	1.16 (-0.86, 3.57)	1.8 (-0.49, 4.59)	2.04 (-0.26, 4.84)	2.04 (-0.5, 4.93)	1.58 (-0.4, 3.97)	1.32 (-0.75, 3.73)	1.68 (-0.36, 4.11)	1.79 (-0.26, 4.22)	1.56 (-0.5, 3.95)	2.13 (0.16, 4.51)	2.07 (0, 4.52)	1.71 (-0.36, 4.1)	1.74 (-0.32, 4.14)	1.32 (-0.57, 3.67)	1.35 (-0.69, 3.72)	-0.14 (-2.68, 2.61)	-0.85 (-4.85, 2.37)	1.51 (-0.63, 4.06)	2.28 (-0.23, 5.21)	3.15 (1.28, 5.59)	1.39 (-0.26, 3.29)	1.55 (-0.7, 4.08)	**29**			
3.08 (0.74, 5.65)	1.71 (-0.74, 4.41)	2.89 (0.4, 5.68)	2.87 (0.39, 5.59)	3.47 (0.92, 6.24)	2.16 (-0.41, 4.9)	2.03 (-0.52, 4.81)	2.67 (-0.08, 5.74)	2.93 (0.17, 6)	2.91 (-0.06, 6.07)	2.46 (-0.09, 5.19)	2.21 (-0.4, 4.97)	2.57 (-0.02, 5.32)	2.67 (0.12, 5.4)	2.44 (-0.16, 5.15)	3.01 (0.5, 5.8)	2.95 (0.37, 5.76)	2.59 (-0.03, 5.38)	2.63 (0, 5.4)	2.22 (-0.25, 4.93)	2.22 (-0.29, 5)	0.72 (-2.19, 3.78)	0.02 (-4.17, 3.51)	2.38 (-0.26, 5.31)	3.15 (0.17, 6.38)	4.03 (1.61, 6.89)	2.26 (0.51, 4.29)	2.43 (-0.28, 5.25)	0.84 (-0.63, 2.43)	**30**		
1.17 (0.15, 2.16)	**-0.19 (-0.86, 0.5)**	0.99 (0.1, 1.95)	0.96 (0.15, 1.84)	1.56 (0.59, 2.63)	0.24 (-0.72, 1.23)	0.13 (-0.85, 1.11)	0.77 (-0.71, 2.37)	1.02 (-0.41, 2.57)	0.99 (-0.7, 2.85)	0.57 (-0.39, 1.47)	0.3 (-0.75, 1.33)	0.67 (-0.36, 1.67)	0.78 (-0.31, 1.78)	0.55 (-0.59, 1.52)	1.1 (0.15, 2.16)	1.06 (-0.03, 2.17)	0.68 (-0.33, 1.69)	0.72 (-0.28, 1.72)	0.31 (-0.41, 1.08)	0.32 (-0.38, 1.03)	**-1.16 (-2.97, 0.55)**	**-1.8 (-5.56, 0.47)**	0.49 (-0.77, 1.76)	1.22 (-0.51, 3.15)	2.15 (0.28, 4.08)	0.36 (-2.46, 3.09)	0.52 (-1.21, 2.19)	-1.01 (-3.42, 1)	**-1.89 (-4.69, 0.65)**	**31**	
0.96 (-0.04, 1.92)	**-0.39 (-1.06, 0.28)**	0.78 (-0.1, 1.73)	0.75 (-0.05, 1.62)	1.35 (0.39, 2.42)	0.04 (-0.92, 1.02)	**-0.07 (-1.05, 0.9)**	0.56 (-0.9, 2.17)	0.8 (-0.6, 2.39)	0.78 (-0.9, 2.64)	0.36 (-0.57, 1.26)	0.08 (-0.96, 1.12)	0.45 (-0.55, 1.45)	0.57 (-0.51, 1.57)	0.34 (-0.79, 1.29)	0.9 (-0.06, 1.93)	0.84 (-0.24, 1.95)	0.48 (-0.52, 1.47)	0.51 (-0.48, 1.5)	**0.1 (-0.6, 0.86)**	**0.11 (-0.58, 0.81)**	**-1.38 (-3.18, 0.32)**	**-2.03 (-5.75, 0.25)**	0.27 (-0.98, 1.56)	1.02 (-0.7, 2.95)	1.94 (0.05, 3.86)	0.14 (-2.65, 2.88)	0.31 (-1.39, 1.96)	**-1.23 (-3.62, 0.78)**	**-2.11 (-4.87, 0.42)**	**-0.21 (-0.91, 0.5)**	**32**

B. The network meta-analysis results of effective rate comparisons.

Note: Comparisons between treatments should be read from left to right, and the estimate is in the cell in common between the column-defining treatment

and the row-defining treatment. The lower the efficacy value after treatment, the more significant the efficacy of the column. Significant results are set in boldface.

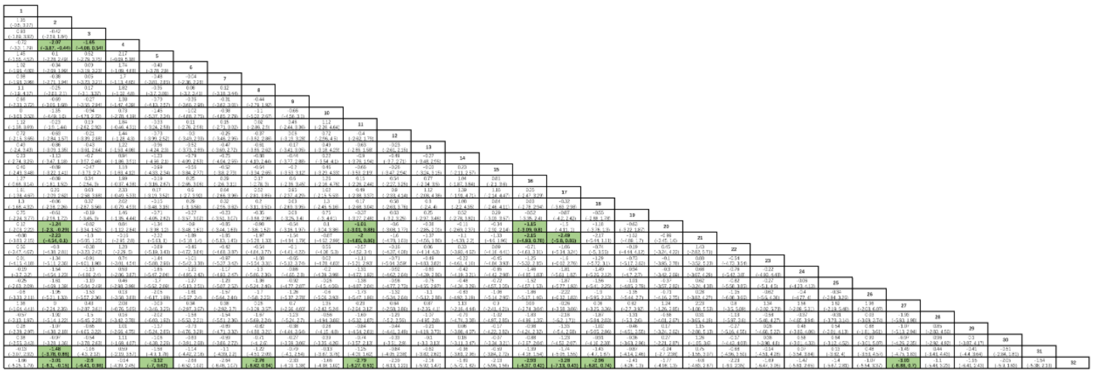

C. The network meta-analysis results of adverse events comparisons.

Note: Comparisons between treatments should be read from left to right, and the estimate is in the cell in common between the column-defining treatment and the row-defining treatment. The lower the efficacy value after treatment, the lower the incidence of adverse reactions in the column. Significant results are set in boldface
