# Supplementary material for: Efficacy and safety of different medications compared for the treatment of postherpetic neuralgia: a network meta-analysis
Source: Front Pharmacol. 2025 Jul 30;16:1614587. doi: 10.3389/fphar.2025.1614587 (PMC12343574; doi:10.3389/fphar.2025.1614587)

## Comparison-adjusted funnel plots

### A. The Results of the Pain Scores

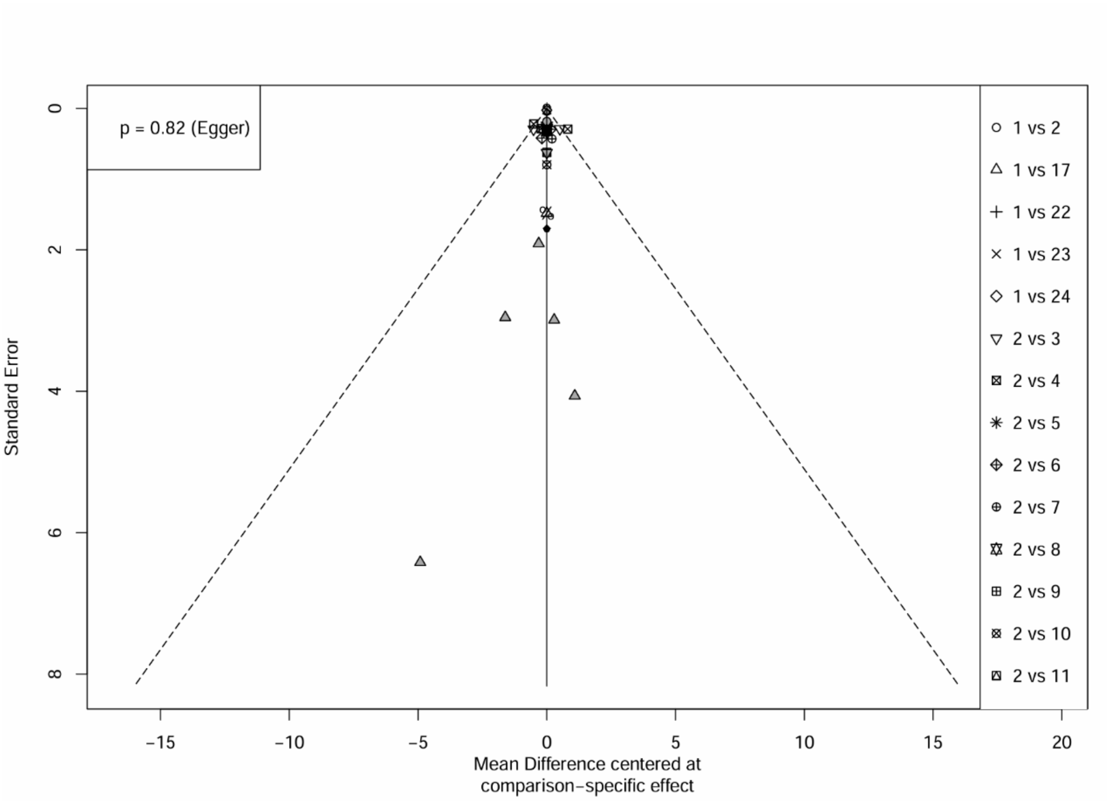

## B. The Results of the Effective Rate

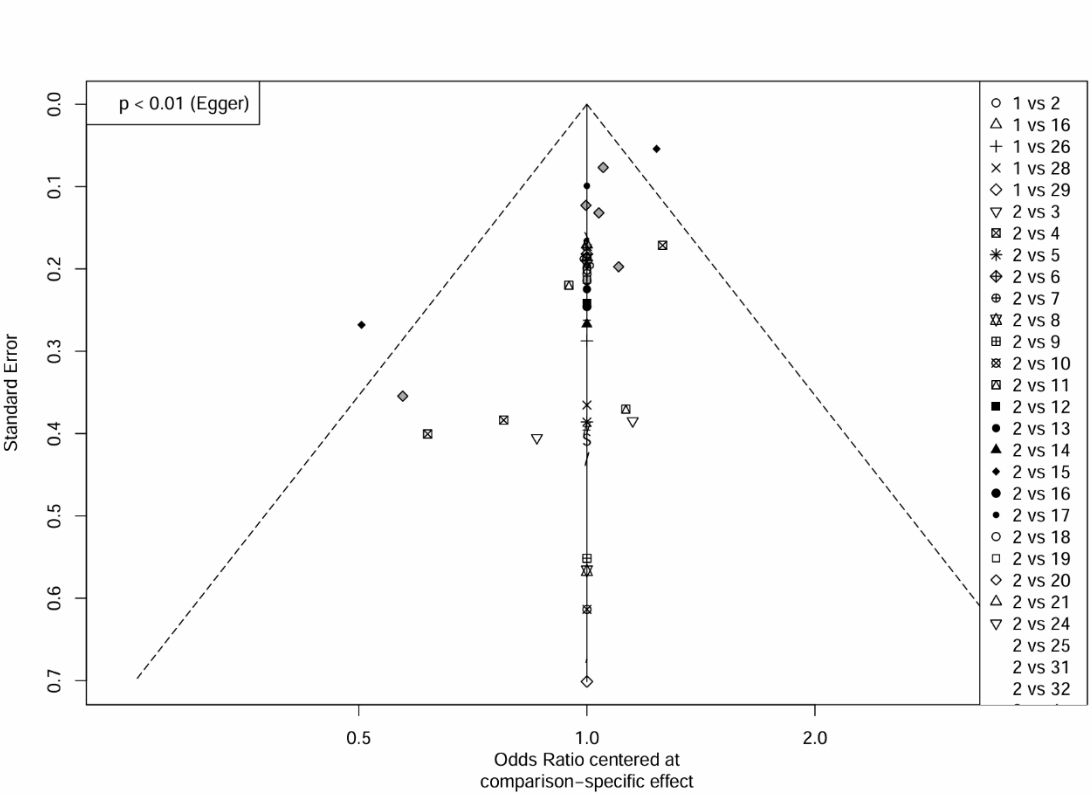


## C. The Results of the Adverse Events

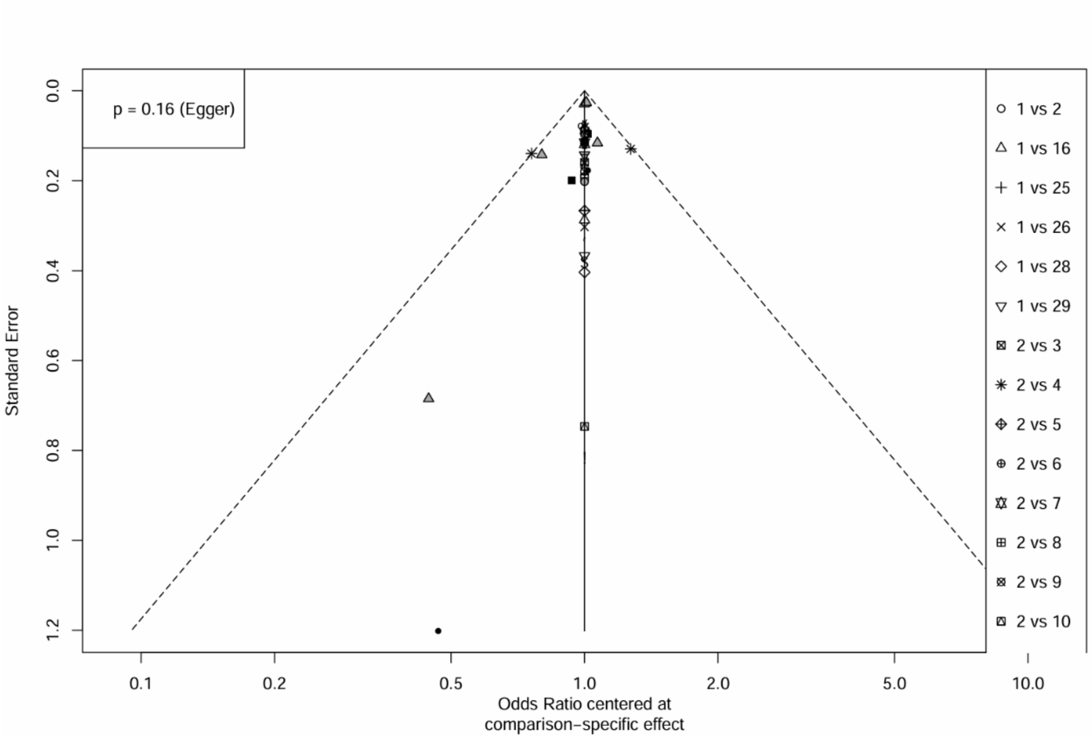

Supplement: Supplementary file 3 [file DataSheet6.pdf]
