# Supplementary material for: Efficacy and safety of different medications compared for the treatment of postherpetic neuralgia: a network meta-analysis
Source: Front Pharmacol. 2025 Jul 30;16:1614587. doi: 10.3389/fphar.2025.1614587 (PMC12343574; doi:10.3389/fphar.2025.1614587)

Forest plots of the network meta-analyses

A. The Results of the Pain Scores

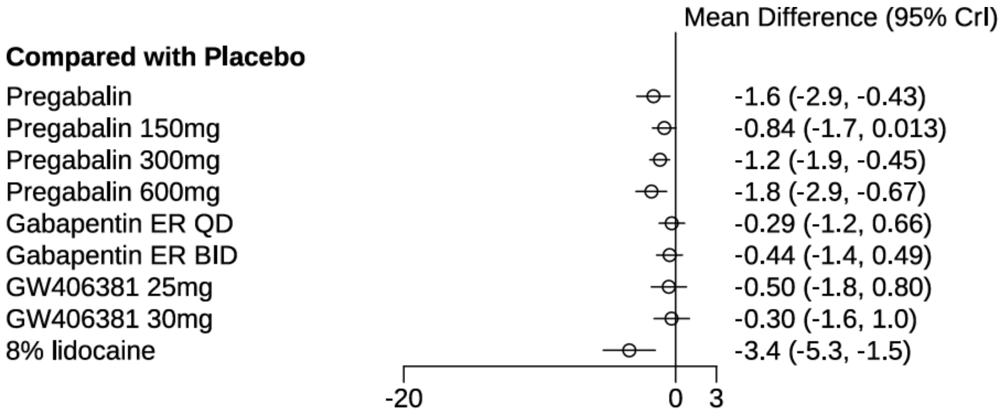

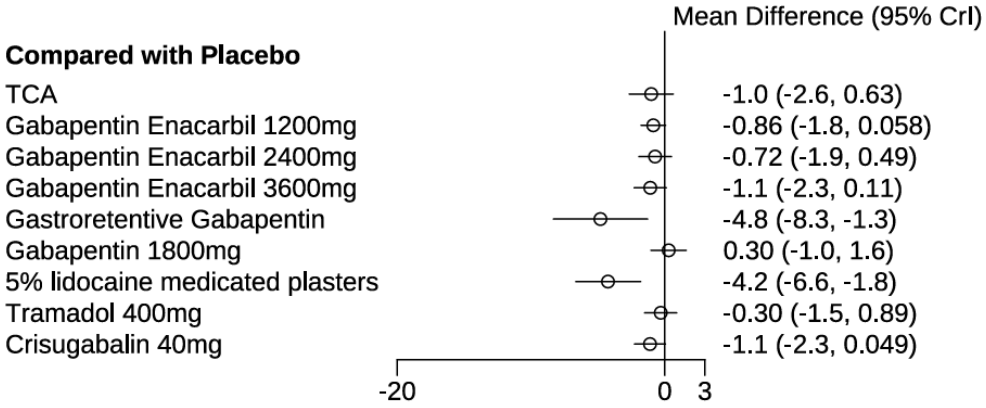

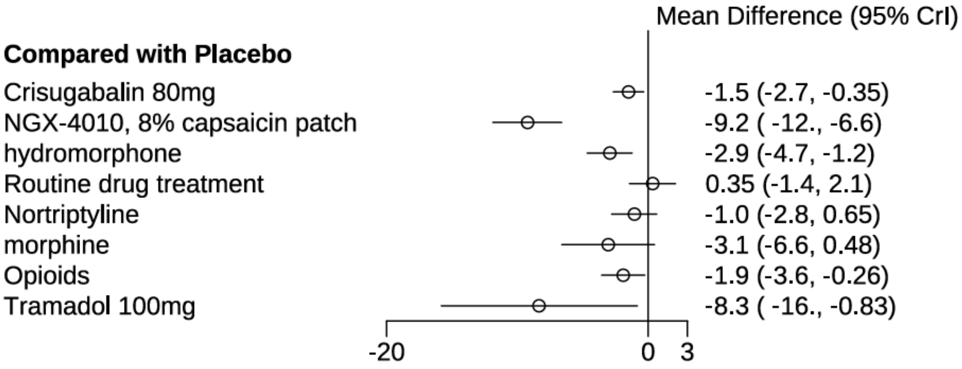

B. The Results of the SFMPQ Scores

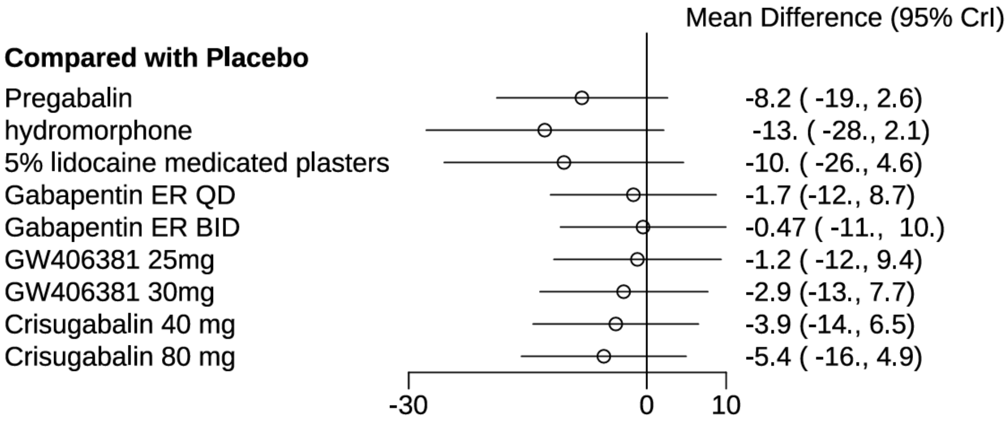

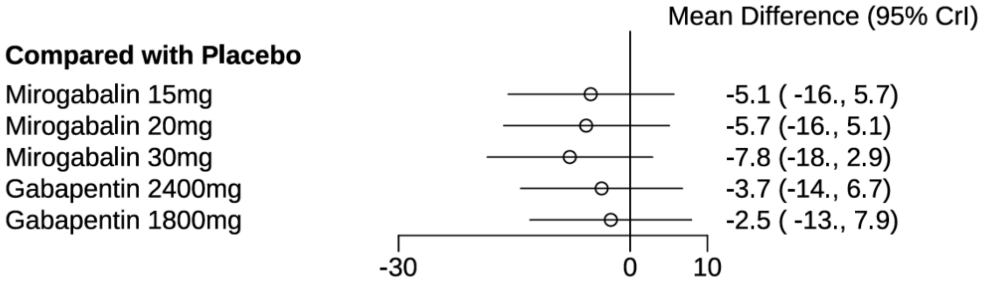

C. The Results of the PSQI Scores

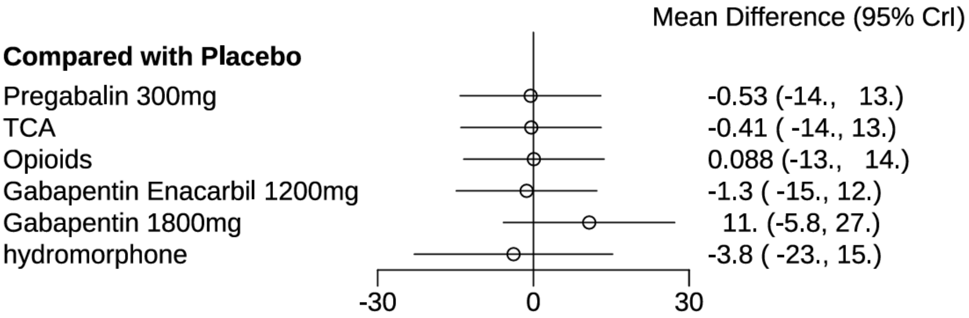

D. The Results of the Effective Rate

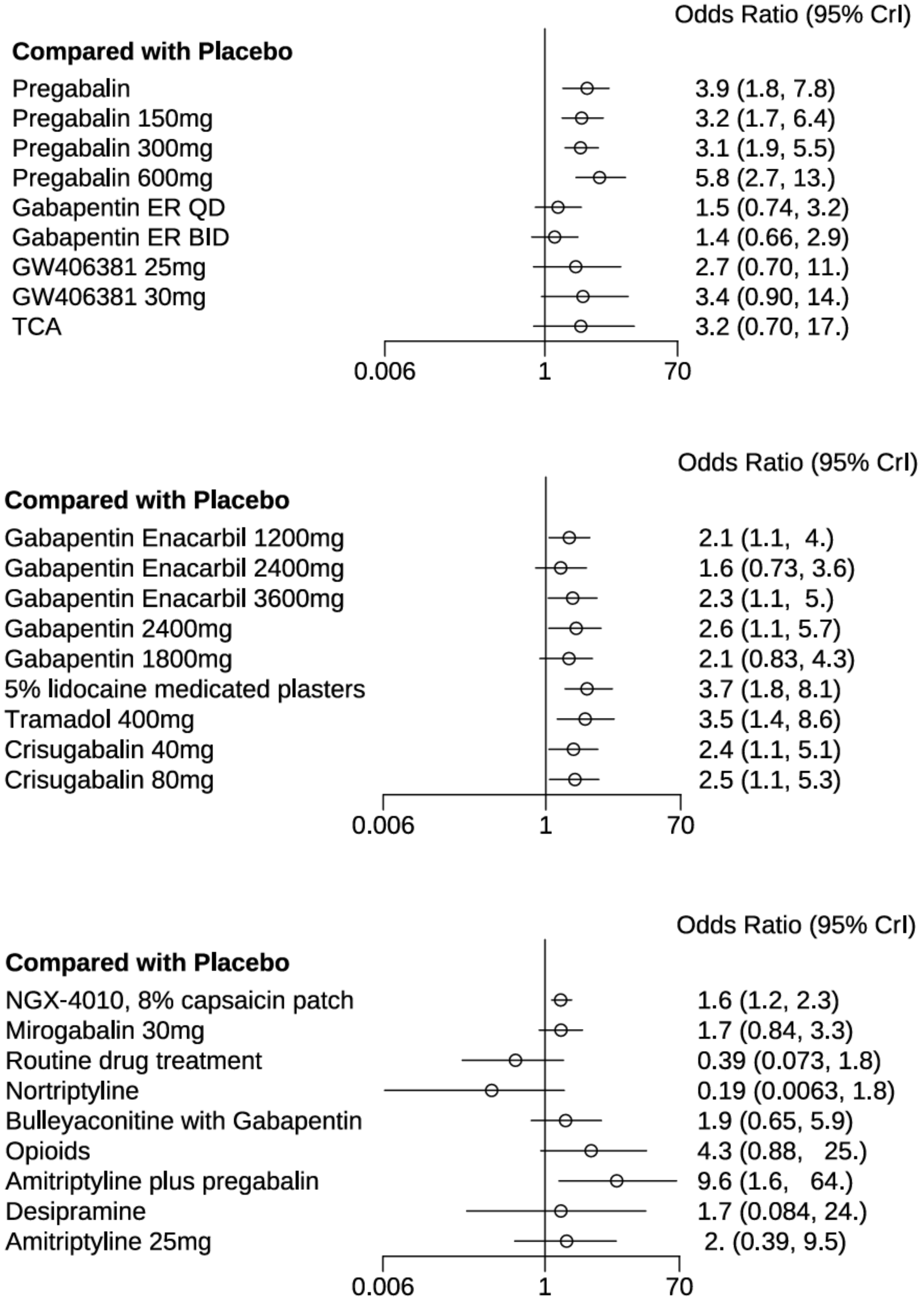

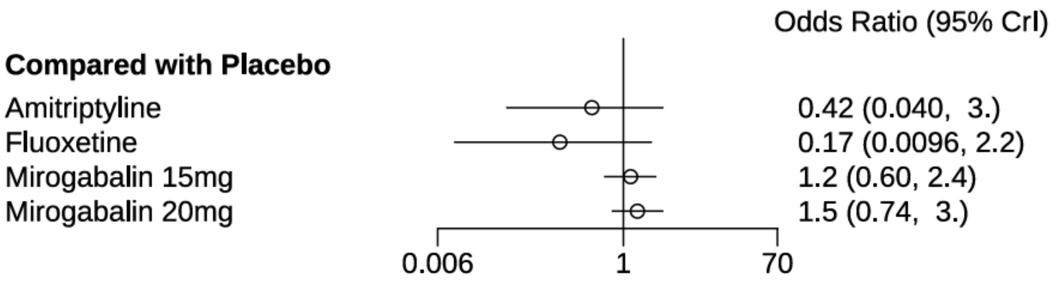

E. The Results of the Adverse Events

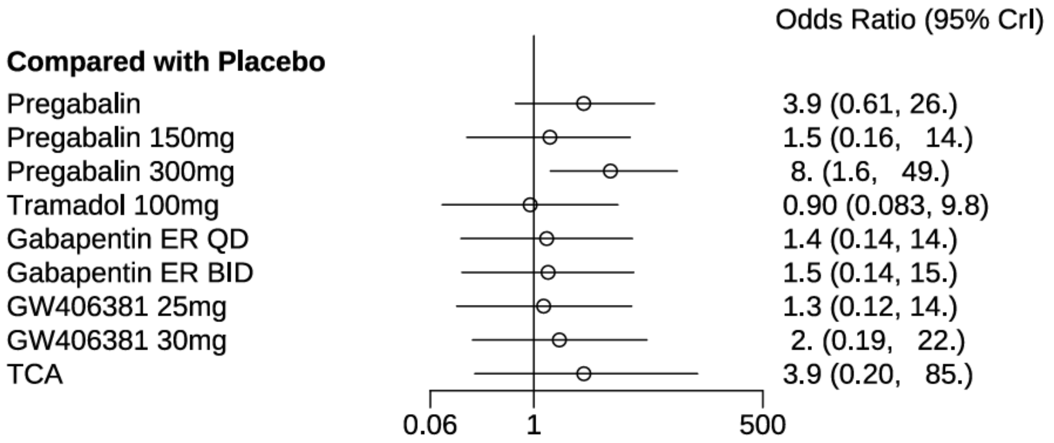

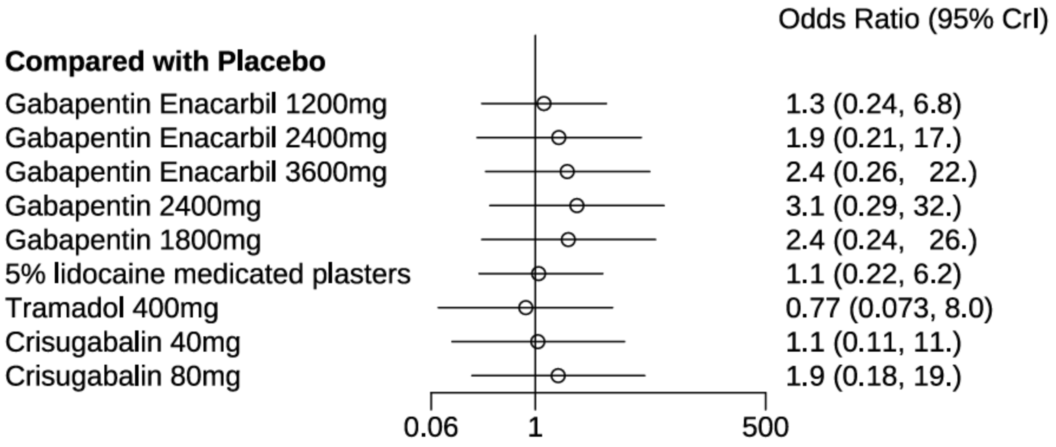

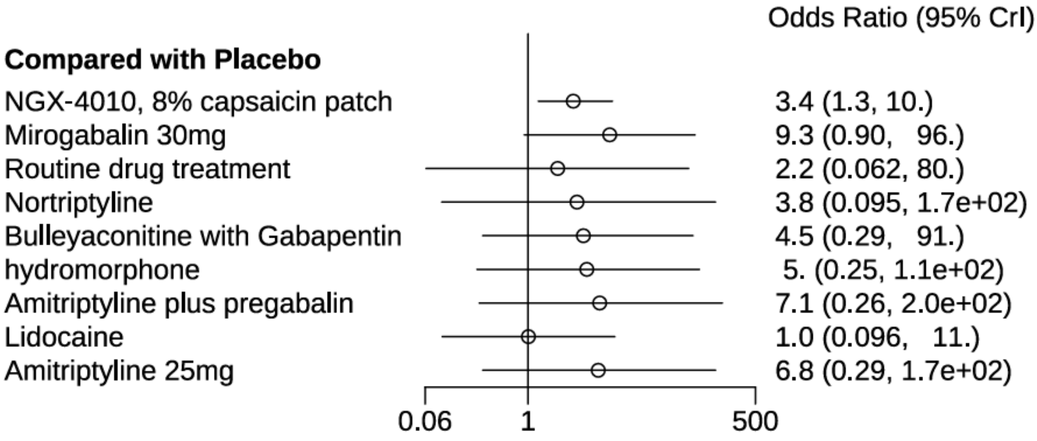
Odds Ratio (95% CrI)
Compared with Placebo
NGX-4010, 8% capsaicin patch
Mirogabalin 30mg
Routine drug treatment
Nortriptyline
Bulleyaconitine with Gabapentin
hydromorphone
Amitriptyline plus pregabalin
Lidocaine
Amitriptyline 25mg
3.4 (1.3, 10.)
9.3 (0.90, 96.)
2.2 (0.062, 80.)
3.8 (0.095, 1.7e+02)
4.5 (0.29, 91.)
5. (0.25, 1.1e+02)
7.1 (0.26, 2.0e+02)
1.0 (0.096, 11.)
6.8 (0.29, 1.7e+02)
0.06
1
500

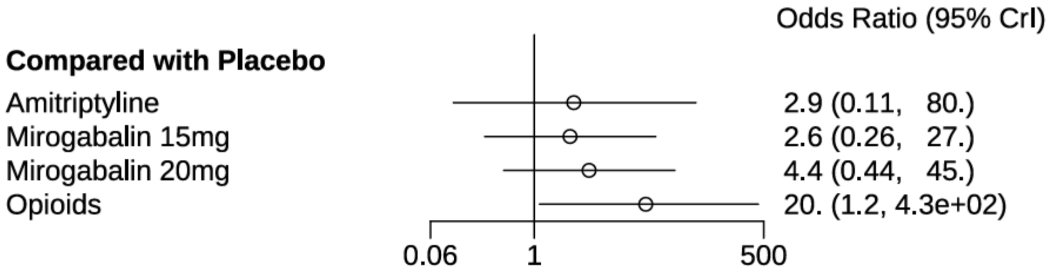
Odds Ratio (95% CrI)
Compared with Placebo
Amitriptyline
Mirogabalin 15mg
Mirogabalin 20mg
Opioids
2.9 (0.11, 80.)
2.6 (0.26, 27.)
4.4 (0.44, 45.)
20. (1.2, 4.3e+02)
0.06
1
500

F. The Network meta-regression analysis results of adverse events

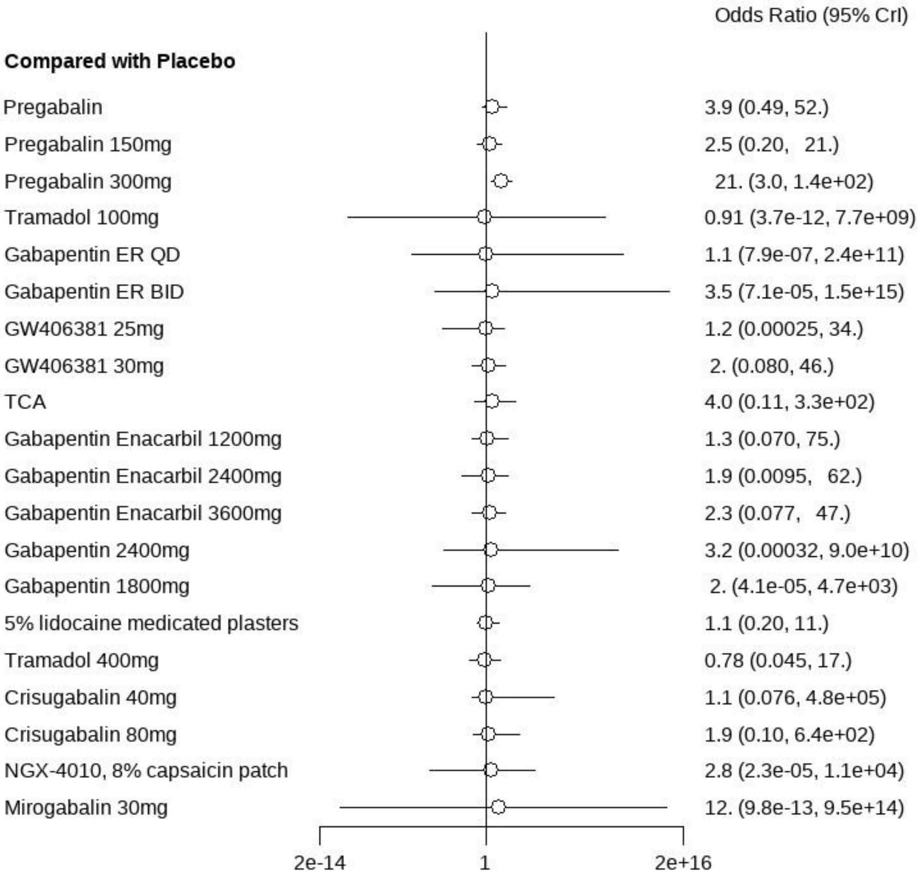

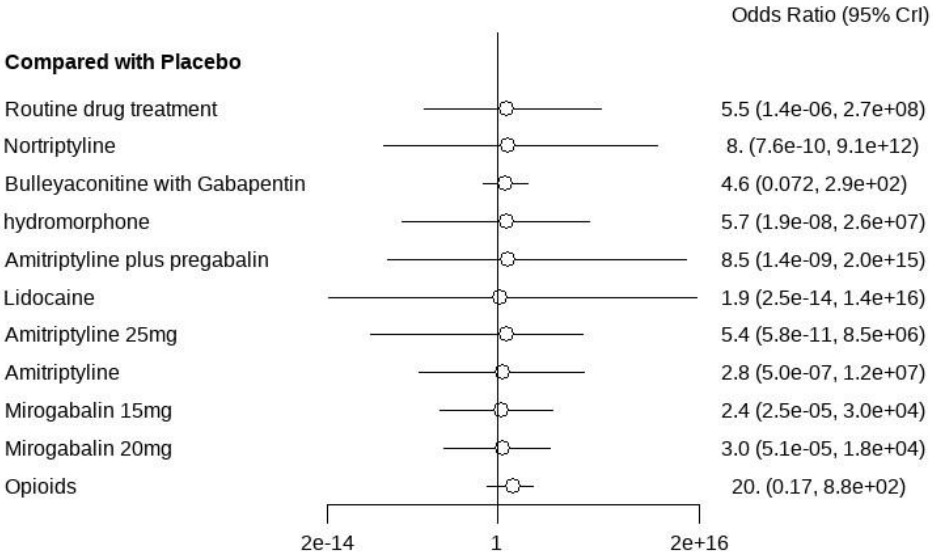

Supplement: Supplementary file 4 [file DataSheet3.pdf]
