# Supplementary material for: Efficacy and safety of different medications compared for the treatment of postherpetic neuralgia: a network meta-analysis
Source: Front Pharmacol. 2025 Jul 30;16:1614587. doi: 10.3389/fphar.2025.1614587 (PMC12343574; doi:10.3389/fphar.2025.1614587)
Supplement: Supplementary file 5 [file DataSheet1.pdf]

Search Strategy

Pubmed

Query	Search term
#1	MeSH terms: “Neuralgia, Postherpetic” OR “Herpes Zoster”
#2	Title/abstract: “postherpetic neuralgia” OR “post-herpetic neuralgia” OR “PHN” OR “herpes zoster” OR “zona” OR “zoster” OR “shingles”
#3	#1 OR #2
#4	MeSH terms: “Drug therapy”
#5	Title/abstract: “Pregabalin” OR “Gabapentin” OR” Lidocaine Patches” OR “Capsaicin Creams” OR” Opioids” OR “Tramadol” OR “Amitriptyline” OR “Desipramine”
#6	#4 OR #5
#7	Publication type: "randomized controlled trial" OR "controlled clinical trial
#8	Title/abstract "randomized" OR "placebo" OR "trial" OR "randomly" OR "groups"
#9	#7 OR #8
#10	#3 AND #6 AND #9

EMBASE

Query	Search term
#1	MeSH terms: “postherpetic neuralgia” OR “herpes zoster”
#2	Title/abstract: “herpetic neuralgia” OR “neuralgia, postherpetic” OR “postherpetic pain” OR “postherpetic neuralgia”
#3	#1 OR #2
#4	MeSH terms: “drug therapy” OR “pregabalin” OR “gabapentin” OR “lidocaine” OR “capsaicin” OR “opioid use” OR “tramadol” OR “amitriptyline” OR “desipramine”
#5	Title/abstract: “drug therapy” OR “drug treatment” OR “medicament therapy” OR “medicament treatment” OR “medication” OR “medicinal intervention” OR “medicinal therapy” OR “medicinal treatment” OR “pharmaceutic intervention” OR “pharmaceutical intervention” OR “pharmaceutical therapy” OR “pharmaceutical treatment” OR “pharmaco therapy” OR “pharmaco treatment” OR “pharmacologic intervention” OR “pharmacological intervention” OR “pharmacological therapy” OR “pharmacological treatment” OR “pharmacotherapy” OR “pharmacotreatment” OR “therapeutic uses” OR “therapy, drug” OR “therapy, pharmacological” OR “treatment, drug” OR “treatment, pharmacological”
#6	#4 OR #5
#7	MeSH terms: “randomized controlled trial”

#8	Title/abstract: “controlled trial, randomized” OR “randomised controlled study” OR “randomised controlled trial” OR “randomized controlled study” OR “trial, randomized controlled” OR “randomized controlled trial”
#9	#7 OR #8
#10	#3 AND #6 AND #9

Web of Science

Query	Search term
#1	((((((((TS=(Neuralgia, Postherpetic)) OR TS=(Herpes Zoster)) OR AB=(postherpetic neuralgia)) OR AB=(post-herpetic neuralgia)) OR AB=(PHN)) OR AB=(herpes zoster)) OR AB=(zona)) OR AB=(zoster)) OR AB=(shingles)
#2	((((((((TS=(Drug Therapy)) OR AB=(Pregabalin)) OR AB=(Gabapentin)) OR AB=(Lidocaine Patches)) OR AB=(Capsaicin Creams)) OR AB=(Opioids)) OR AB=(Tramadol)) OR AB=(Amitriptyline)) OR AB=(Desipramine)
#3	((((((TS=(randomized controlled trial)) OR TS=(controlled clinical trial)) OR AB=(randomized)) OR AB=(placebo)) OR AB=(trial)) OR AB=(randomly)) OR AB=(groups)
#4	#1 AND #2 AND #3

Medline

Query	Search term
#1	((((((((TS=(Neuralgia, Postherpetic)) OR TS=(Herpes Zoster)) OR AB=(postherpetic neuralgia)) OR AB=(post-herpetic neuralgia)) OR AB=(PHN)) OR AB=(herpes zoster)) OR AB=(zona)) OR AB=(zoster)) OR AB=(shingles)
#2	((((((((TS=(Drug Therapy)) OR AB=(Pregabalin)) OR AB=(Gabapentin)) OR AB=(Lidocaine Patches)) OR AB=(Capsaicin Creams)) OR AB=(Opioids)) OR AB=(Tramadol)) OR AB=(Amitriptyline)) OR AB=(Desipramine)
#3	((((((TS=(randomized controlled trial)) OR TS=(controlled clinical trial)) OR AB=(randomized)) OR AB=(placebo)) OR AB=(trial)) OR AB=(randomly)) OR AB=(groups)
#4	#1 AND #2 AND #3

The Cochrane Library

Query	Search term
#1	MeSH descriptor: [Neuralgia, Postherpetic]

#2	MeSH descriptor: [Herpes Zoster]
#3	#1 OR #2
#4	Title/abstract: (postherpetic neuralgia OR post-herpetic neuralgia OR PHN OR herpes zoster OR zona OR zoster OR shingles)
#5	#3 OR #4
#6	MeSH descriptor: [Drug Therapy]
#7	Title/abstract: (Pregabalin OR Gabapentin OR Lidocaine Patches OR Capsaicin Creams OR Opioids OR Tramadol OR Amitriptyline OR Desipramine)
#8	#6 OR #7
#9	#5 AND #8
