# Supplementary material for: Efficacy and safety of different medications compared for the treatment of postherpetic neuralgia: a network meta-analysis
Source: Front Pharmacol. 2025 Jul 30;16:1614587. doi: 10.3389/fphar.2025.1614587 (PMC12343574; doi:10.3389/fphar.2025.1614587)
Supplement: Supplementary file 6 [file DataSheet5.pdf]

Cumulative probability plots

(the Surface Under the Cumulative Ranking Area)

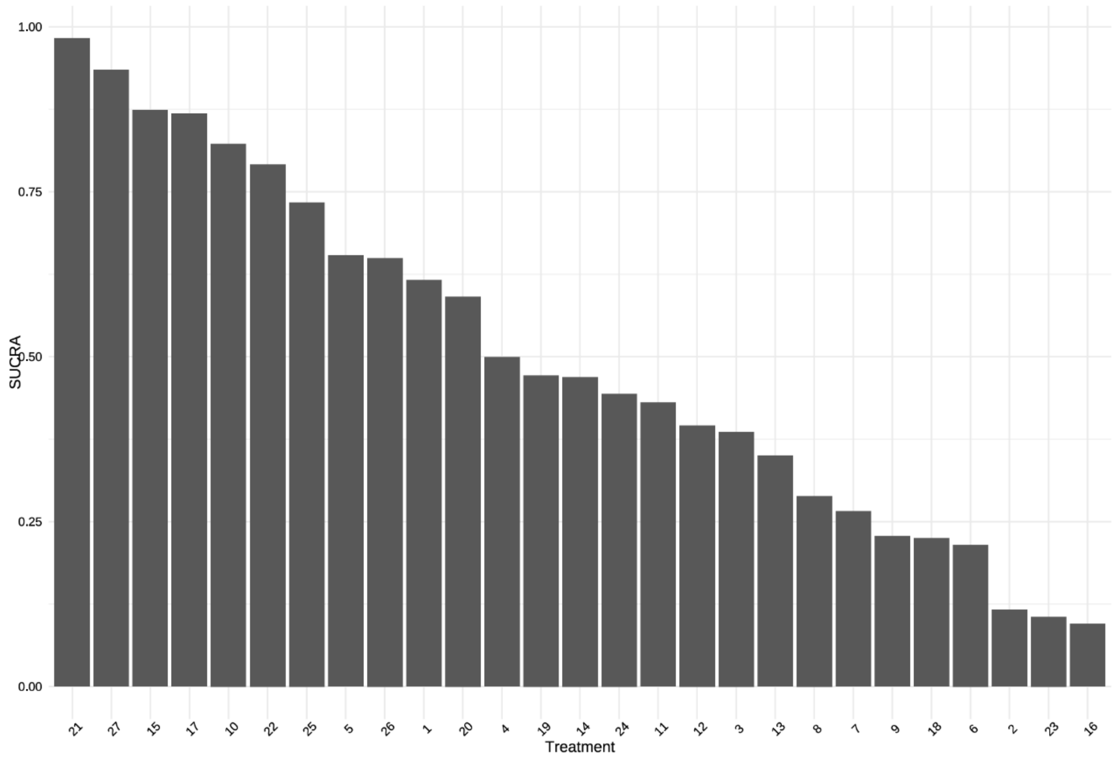

A. **The results of pain scores, 21= NGX-4010, 8% capsaicin patch,27= Tramadol 100mg,15= Gastroretentive Gabapentin.**

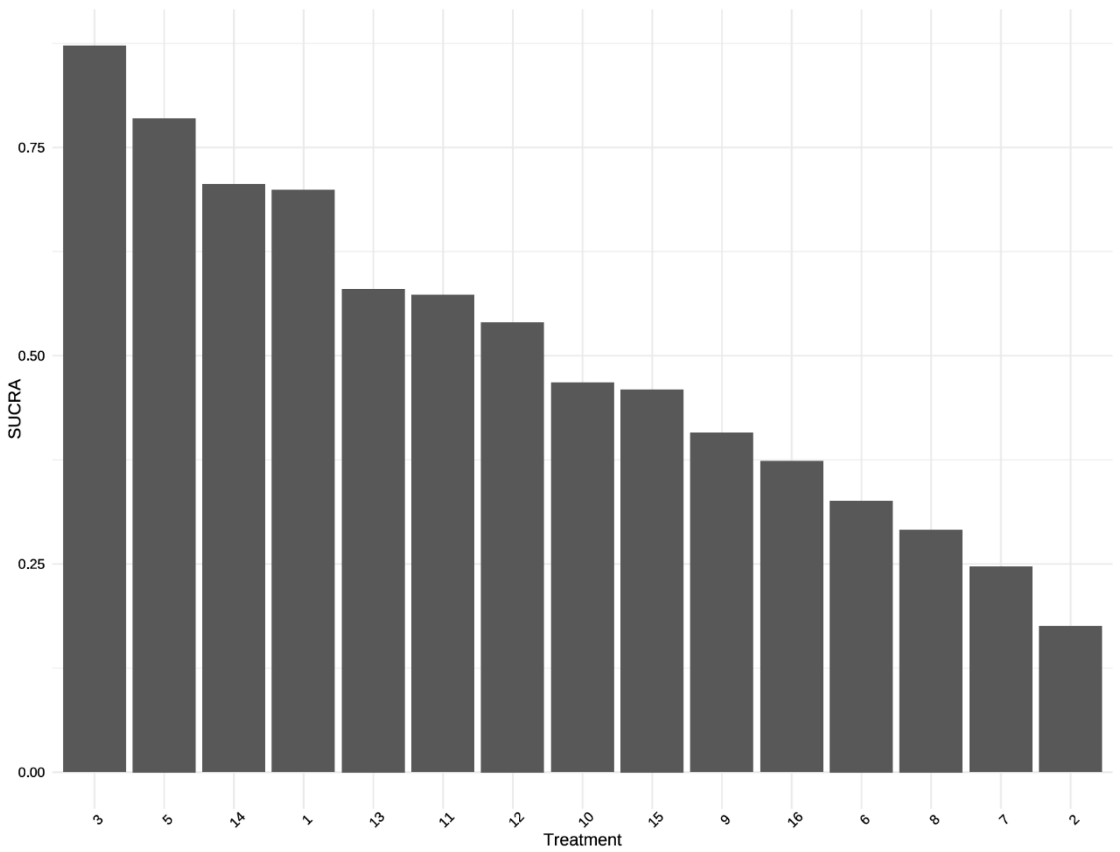

B. **The results of SFMPQ scores, 3= hydromorphone, 5= 5% lidocaine medicated plasters, 14= Mirogabalin 30mg.**

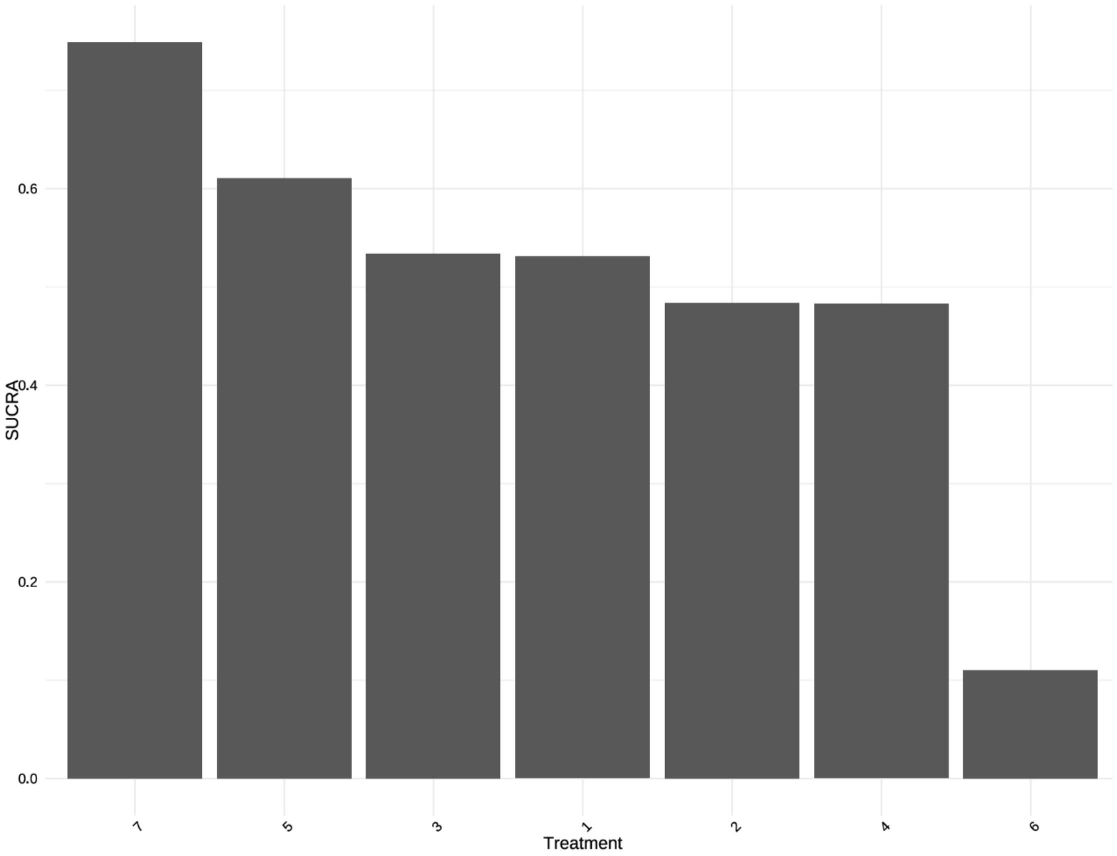

C. **The results of PSQI scores,7= hydromorphone,5= Gabapentin Enacarbil 1200mg,3= TCAs.**

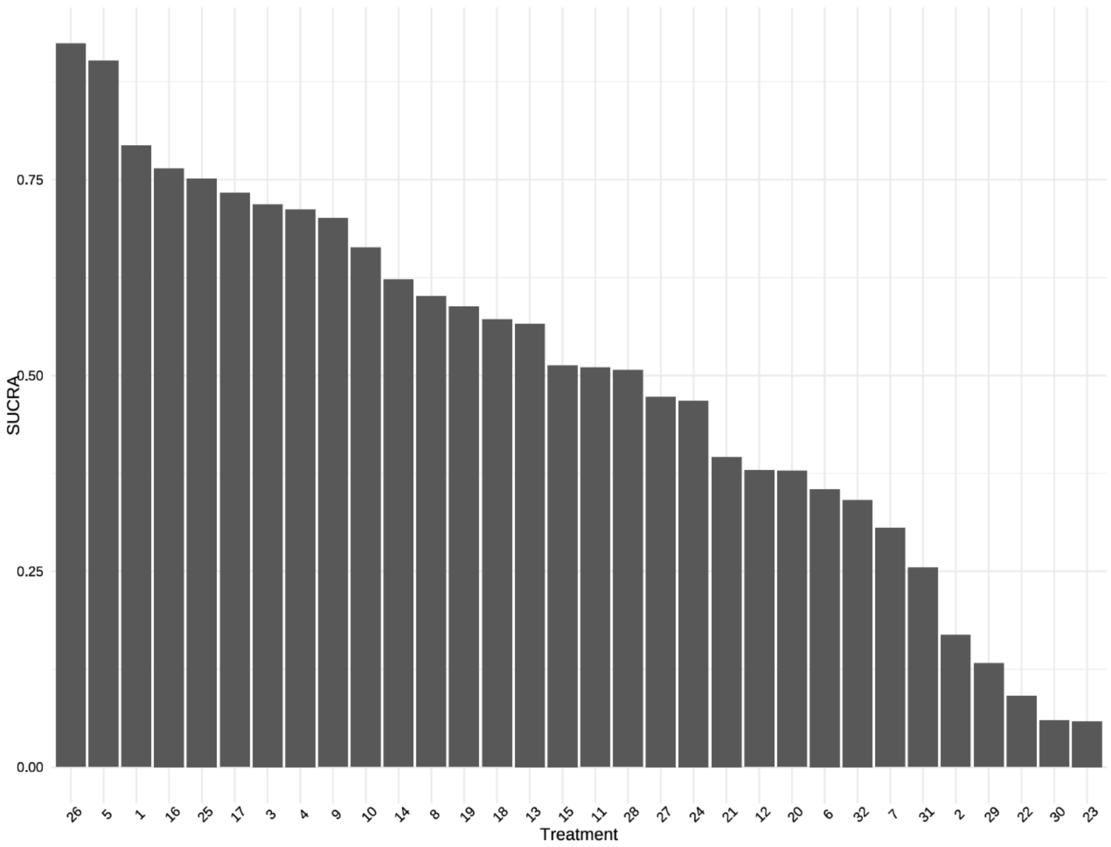

D. **The results of effective rate,26= Amitriptyline plus pregabalin,5= Pregabalin 600mg,1= Pregabalin.**

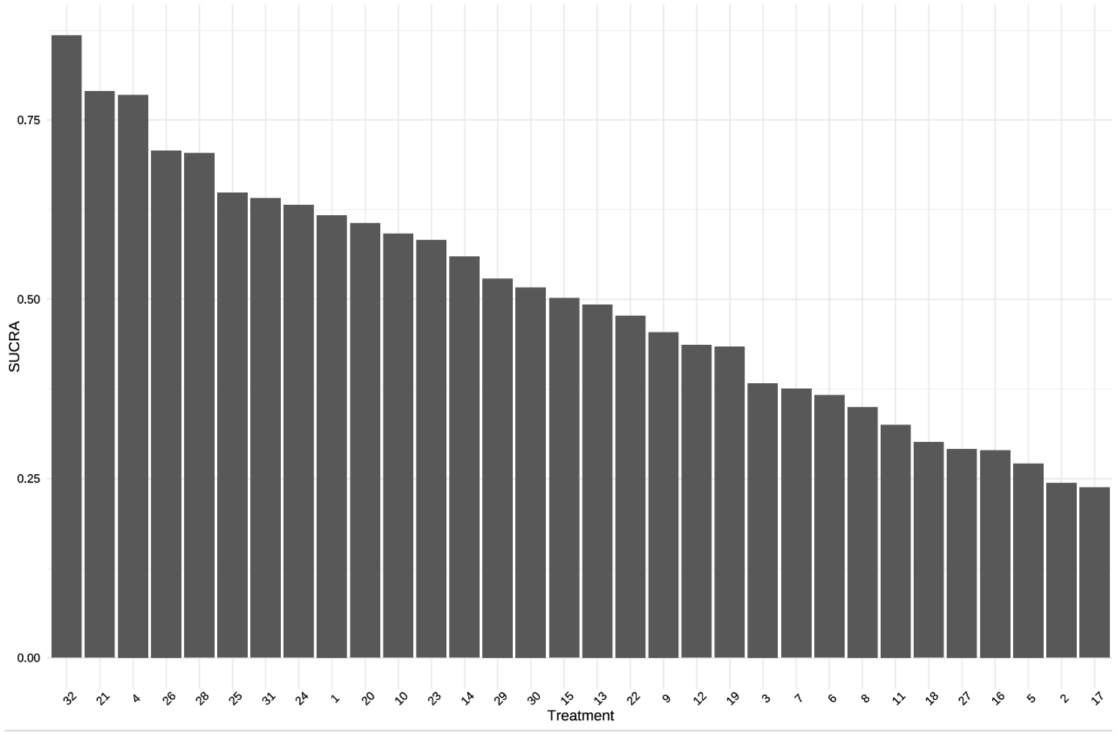

E. The results of AEs,32= Opioids,21= Mirogabalin 30mg, 4= Pregabalin 300mg.
